# Plant-Based Dietary Patterns and Incidence of Type 2 Diabetes in US Men and Women: Results from Three Prospective Cohort Studies

**DOI:** 10.1371/journal.pmed.1002039

**Published:** 2016-06-14

**Authors:** Ambika Satija, Shilpa N. Bhupathiraju, Eric B. Rimm, Donna Spiegelman, Stephanie E. Chiuve, Lea Borgi, Walter C. Willett, JoAnn E. Manson, Qi Sun, Frank B. Hu

**Affiliations:** 1 Department of Nutrition, Harvard T.H. Chan School of Public Health, Boston, Massachusetts, United States of America; 2 Department of Epidemiology, Harvard T.H. Chan School of Public Health, Boston, Massachusetts, United States of America; 3 Channing Division of Network Medicine, Department of Medicine, Brigham and Women’s Hospital, Boston, Massachusetts, United States of America; 4 Department of Biostatistics, Harvard T.H. Chan School of Public Health, Boston, Massachusetts, United States of America; 5 Department of Global Health, Harvard T.H. Chan School of Public Health, Boston, Massachusetts, United States of America; 6 Division of Preventive Medicine, Department of Medicine, Brigham and Women’s Hospital, Boston, Massachusetts, United States of America; 7 Renal Division, Brigham and Women’s Hospital, Boston, Massachusetts, United States of America; 8 Harvard Medical School, Boston, Massachusetts, United States of America; National Cancer Institute, UNITED STATES

## Abstract

**Background:**

Plant-based diets have been recommended to reduce the risk of type 2 diabetes (T2D). However, not all plant foods are necessarily beneficial. We examined the association of an overall plant-based diet and hypothesized healthful and unhealthful versions of a plant-based diet with T2D incidence in three prospective cohort studies in the US.

**Methods and Findings:**

We included 69,949 women from the Nurses’ Health Study (1984–2012), 90,239 women from the Nurses’ Health Study 2 (1991–2011), and 40,539 men from the Health Professionals Follow-Up Study (1986–2010), free of chronic diseases at baseline. Dietary data were collected every 2–4 y using a semi-quantitative food frequency questionnaire. Using these data, we created an overall plant-based diet index (PDI), where plant foods received positive scores, while animal foods (animal fats, dairy, eggs, fish/seafood, poultry/red meat, miscellaneous animal-based foods) received reverse scores. We also created a healthful plant-based diet index (hPDI), where healthy plant foods (whole grains, fruits, vegetables, nuts, legumes, vegetable oils, tea/coffee) received positive scores, while less healthy plant foods (fruit juices, sweetened beverages, refined grains, potatoes, sweets/desserts) and animal foods received reverse scores. Lastly, we created an unhealthful plant-based diet index (uPDI) by assigning positive scores to less healthy plant foods and reverse scores to healthy plant foods and animal foods.

We documented 16,162 incident T2D cases during 4,102,369 person-years of follow-up. In pooled multivariable-adjusted analysis, both PDI and hPDI were inversely associated with T2D (PDI: hazard ratio [HR] for extreme deciles 0.51, 95% CI 0.47–0.55, *p* trend < 0.001; hPDI: HR for extreme deciles 0.55, 95% CI 0.51–0.59, *p* trend < 0.001). The association of T2D with PDI was considerably attenuated when we additionally adjusted for body mass index (BMI) categories (HR 0.80, 95% CI 0.74–0.87, *p* trend < 0.001), while that with hPDI remained largely unchanged (HR 0.66, 95% CI 0.61–0.72, *p* trend < 0.001). uPDI was positively associated with T2D even after BMI adjustment (HR for extreme deciles 1.16, 95% CI 1.08–1.25, *p* trend < 0.001). Limitations of the study include self-reported diet assessment, with the possibility of measurement error, and the potential for residual or unmeasured confounding given the observational nature of the study design.

**Conclusions:**

Our study suggests that plant-based diets, especially when rich in high-quality plant foods, are associated with substantially lower risk of developing T2D. This supports current recommendations to shift to diets rich in healthy plant foods, with lower intake of less healthy plant and animal foods.

## Abstract 2

Ambika Satija and colleagues study associations between plant-based diet indices and type 2 diabetes incidence in large prospective cohorts.

## Author Summary

**Why Was This Study Done?:**

- Plant-based diets, mainly defined as “vegetarian” diets, have been associated with improved health outcomes, including reduced risk of diabetes.
- However, vegetarian diets can include less healthy plant foods, such as sweetened foods and beverages, which are detrimental for health. Also, as it would be difficult for many individuals to completely give up some or all animal foods to become vegetarian, it is important to understand how gradually increasing plant foods, while decreasing animal foods, affects diabetes risk.
- Thus, in this study, we aimed to understand how gradations of adherence to different types of plant-based diets (healthful and unhealthful) are associated with diabetes risk.

**What Did the Researchers Do and Find?:**

- We analyzed data from three studies that followed more than 200,000 male and female health professionals across the US for more than 20 y, regularly collecting information on their diet, lifestyle, medical history, and new disease diagnoses.
- We found that having a diet that emphasized plant foods and was low in animal foods was associated with a reduction of about 20% in the risk of diabetes.
- Consumption of a plant-based diet that emphasized specifically healthy plant foods was associated with a larger decrease (34%) in diabetes risk, while consumption of a plant-based diet high in less healthy plant foods was associated with a 16% increased diabetes risk.

**What Do These Findings Mean?:**

- Increasing intake of healthy plant foods while moderately reducing intake of some animal foods, especially red and processed meats, may be beneficial for diabetes prevention.
- These findings support the newly released 2015–2020 Dietary Guidelines for Americans.

## Introduction

Type 2 diabetes (T2D) is associated with increased morbidity, mortality, and healthcare costs in the US [1]. Several plant foods, such as whole grains, fruits, and vegetables, are associated with a lower risk of T2D [2–4], while certain animal foods, such as red and processed meats, are positively associated with T2D risk [5]. Additionally, the recently released 2015 Dietary Guidelines Advisory Committee report recommends shifting away from intake of certain animal foods and moving towards a plant-rich diet [6]. Thus, we evaluated the hypothesis that a plant-based diet is protective against T2D.

Prior studies on plant-based diets and T2D [7–9] have defined plant-based diets as “vegetarian” diets, categorizing study populations dichotomously into participants who do or do not consume some or all animal foods. An important question from clinical and public health standpoints, however, is whether gradually moving towards a plant-rich diet by progressively decreasing animal food intake lowers T2D risk. If so, public health recommendations could suggest incremental dietary changes. Existing studies of vegetarian diets and T2D are also limited by a lack of differentiation among plant foods with divergent effects on T2D, because less nutrient-dense plant foods, such as refined grains, potatoes, and sugar-sweetened beverages, are associated with higher T2D risk [10–12].

We thus conceptualized a graded dietary pattern that positively weighs plant foods and negatively weighs animal foods, similar to the approach used by Martínez-González et al. [13]. We examined the association of this overall plant-based diet and, a priori, healthful and unhealthful versions of a plant-based diet with T2D incidence in three large prospective cohort studies in the US. We hypothesized that these plant-based diets would be inversely associated with T2D risk.

## Methods

Study protocols for all cohorts were approved by the institutional review boards of Brigham and Women’s Hospital and the Harvard T.H. Chan School of Public Health; completion of the self-administered questionnaire was considered to imply informed consent.

### Study Population

The Nurses’ Health Study (NHS) started in 1976 with 121,701 female nurses (aged 30–55 y) [14], the Nurses’ Health Study 2 (NHS2) started in 1989 with 116,430 female nurses (aged 25–42 y) [15], and the Health Professionals Follow-Up Study (HPFS) started in 1986 with 51,529 male health professionals (aged 40–75 y) [16]; all three studies recruited participants from across the US. In all three studies, follow-up questionnaires collect information on lifestyle and medical history biennially, with a response rate of ~90% per cycle. In the current analysis, the 1984, 1991, and 1986 cycles were the baselines for NHS, NHS2, and HPFS, respectively, because these are the cycles in which data on most covariates of interest were first comprehensively measured. Participants with diabetes, cancer (except nonmelanoma skin cancer), cardiovascular disease (CVD), reported energy intake levels outside predefined limits (<600 or >3,500 kcal/d for women and <800 or >4,200 kcal/d for men), or incomplete dietary data at baseline were excluded. The final analysis included 69,949 women in NHS, 90,239 women in NHS2, and 40,539 men in HPFS at baseline.

### Dietary Assessment

Dietary data were collected every 2–4 y using a semi-quantitative food frequency questionnaire. Participants were asked how often they consumed a defined portion of ~130 food items over the previous year. Response categories ranged from “never or less than once/month” to “≥6 times/day.” The reliability and validity of the questionnaires have been described previously [17–20].

### Plant-Based Diet Indices

We created an overall plant-based diet index (PDI), a healthful plant-based diet index (hPDI), and an unhealthful plant-based diet index (uPDI). The procedure we used to create these indices is similar to the one used by Martínez-González et al. [13]; their “provegetarian food pattern” is similar in composition to our PDI. Frequencies of consumption of each food were converted into servings consumed per day. Then the number of servings of foods that belonged to each of 18 food groups were added up. The 18 food groups were created on the basis of nutrient and culinary similarities, within larger categories of animal foods and healthy and less healthy plant foods. We distinguished between healthy and less healthy plant foods using existing knowledge of associations of the foods with T2D, other outcomes (CVD, certain cancers), and intermediate conditions (obesity, hypertension, lipids, inflammation). Plant foods not clearly associated in one direction with several health outcomes, specifically alcoholic beverages, were not included in the indices. We also excluded margarine from the indices, as its fatty acid composition has changed over time from high trans fat to high unsaturated fat. We controlled for alcoholic beverages and margarine consumption in the analysis.

Healthy plant food groups included whole grains, fruits, vegetables, nuts, legumes, vegetable oils, and tea/coffee, whereas less healthy plant food groups included fruit juices, sugar-sweetened beverages, refined grains, potatoes, and sweets/desserts. Animal food groups included animal fats, dairy, eggs, fish/seafood, meat (poultry and red meat), and miscellaneous animal-based foods.

S1 Table details examples of foods constituting the food groups. The 18 food groups were divided into quintiles of consumption, and each quintile was assigned a score between 1 and 5. For PDI, participants received a score of 5 for each plant food group for which they were above the highest quintile of consumption, a score of 4 for each plant food group for which they were above the second highest quintile but below the highest quintile, and so on, with a score of 1 for consumption below the lowest quintile (positive scores). On the other hand, participants received a score of 1 for each animal food group for which they were above the highest quintile of consumption, a score of 2 for each animal food group for which they were between the highest and second highest quintiles, and so on, with a score of 5 for consumption below the lowest quintile (reverse scores). For hPDI, positive scores were given to healthy plant food groups, and reverse scores to less healthy plant food groups and animal food groups. Finally, for uPDI, positive scores were given to less healthy plant food groups, and reverse scores to healthy plant food groups and animal food groups. The 18 food group scores for an individual were summed to obtain the indices, with a theoretical range of 18 (lowest possible score) to 90 (highest possible score). The observed ranges at baseline were 24–85 (PDI), 28–86 (hPDI), and 27–90 (uPDI) across the cohorts. The indices were analyzed as deciles, with energy intake adjusted at the analysis stage.

### Ascertainment of Type 2 Diabetes

Participants who self-reported physician-diagnosed diabetes were sent a supplementary questionnaire with established validity to confirm diagnosis [21,22]. Only confirmed cases that met ≥1 of the following criteria were included (as per the National Diabetes Data Group) [23]: (a) ≥1 classic symptoms plus fasting blood glucose ≥ 140 mg/dl (>=7.8 mmol/l) or random blood glucose ≥ 200 mg/dl (≥11.1 mmol/l); (b) no symptoms, but raised blood glucose levels (i.e., fasting blood glucose ≥ 140 mg/dl or random blood glucose ≥ 200 mg/dl or 2-h blood glucose after oral glucose tolerance testing ≥ 200 mg/dl) on two different occasions; (c) treatment with hypoglycemic drugs. The threshold for fasting plasma glucose was changed to ≥126 mg/dl (7.0 mmol/l) starting in 1998 [24]. HbA1c ≥ 6.5% was further added to the diagnosis criteria starting in 2010 [25].

### Assessment of Covariates

We collected height at baseline and updated information on weight, physical activity, smoking status, multivitamin use, ethnicity, family history of T2D, hypertension, and hypercholesterolemia through biennial questionnaires. In NHS and NHS2, we also assessed information on menopausal status, postmenopausal hormone use, and oral contraceptive use.

### Statistical Analysis

We calculated person-time for each participant from questionnaire return date until T2D diagnosis, death, censoring, or end of follow-up (30 June 2012 in NHS, 30 June 2011 in NHS2, and 1 January 2010 in HPFS). For the primary analysis, we categorized the indices into deciles, so as to not make assumptions about linearity and to limit the influence of outlying observations. We used Cox proportional-hazards regression to evaluate the associations between deciles of each index and T2D incidence. Age (years) was used as the timescale, with stratification by calendar time (2-y intervals). We adjusted for smoking status, alcohol intake, physical activity, family history of diabetes, multivitamin use, margarine intake, energy intake, baseline hypertension and hypercholesterolemia, body mass index (BMI) categories, menopausal status and postmenopausal hormone use (women), and oral contraceptive use (NHS2). Continuous covariates were included in the model as categories for the reasons cited above for categorizing the indices.

All dietary variables were cumulatively updated, i.e., were averaged, over the entire follow-up duration to better capture long-term diet. Updating was stopped when major outcomes (CVD or cancer) developed, as diagnosis with these conditions could change an individual’s diet. Values of non-dietary covariates were updated every 2 y to account for changes in these variables over time. In order to examine potential nonlinear associations, we created continuous variables of the indices by assigning the median value to each decile and conducting tests for linear trend, examined associations per 10-unit increase in the indices, and used restricted cubic splines. We tested for effect modification by age, physical activity, family history of diabetes, and BMI, by including cross-product terms. The analysis was carried out separately for each cohort, and the cohort-specific HRs were combined using a fixed-effects model; the Cochrane *Q* statistic [26], the *I* ^2^ statistic [27], and the between-study coefficient of variation [28,29] were used to assess heterogeneity among the cohorts. All statistical tests were two-sided (α = 0.05). All analyses were performed using SAS version 9.4 for UNIX (SAS Institute).

## Results

### Baseline Characteristics

The distribution of age-adjusted baseline characteristics according to the PDI and hPDI are shown in Tables S2 and 1, respectively. Participants with higher scores on PDI or hPDI were older, more active, leaner, and less likely to smoke than participants with lower scores. They also consumed a lower percentage of calories from saturated and monounsaturated fats, a higher percentage of calories from polyunsaturated fats and carbohydrates, and higher levels of fiber and folate.

**Table 1:** Age-standardized baseline characteristics by deciles of the healthful plant-based diet index.

Characteristic	NHS (1984)			NHS 2 (1991)			HPFS (1986)		
	Decile 1	Decile 5	Decile 10	Decile 1	Decile 5	Decile 10	Decile 1	Decile 5	Decile 10
**Number of participants**	7,792	7,305	6,608	8,540	9,498	9,439	3,924	4,207	3,604
**Median hPDI**	43	54	67	43	54	67	42	54	67
**hPDI range**	30–48	53–55	63–84	29–47	53–55	62–86	28–47	53–55	63–84
**Age (years)**	48 (6.9)	50 (7.0)	53 (6.7)	35 (4.8)	36 (4.7)	37 (4.4)	50 (8.9)	53 (9.6)	55 (9.2)
**White**	99%	98%	97%	96%	96%	97%	97%	95%	95%
**Current smoker**	28%	25%	19%	14%	12%	10%	13%	9%	5%
**Physical activity (MET-h/wk)**	11 (17)	14 (20)	20 (27)	16 (22)	20 (27)	30 (36)	18 (26)	20 (25)	29 (38)
**BMI (kg/m** ^**2**^ **)**	25 (5.2)	25 (4.5)	24 (4.0)	25 (6.3)	25 (5.2)	24 (4.3)	26 (3.4)	26 (3.2)	25 (3.1)
**Current multivitamin use**	32%	37%	44%	35%	39%	45%	35%	41%	49%
**Premenopausal**	61%	48%	32%	98%	97%	96%	—	—	—
**Current postmenopausal hormone use**	7.7%	11%	16%	1.9%	2.7%	3.3%	—	—	—
**Current oral contraceptive use**	—	—	—	13%	10%	9%	—	—	—
**Family history of diabetes**	28%	28%	28%	35%	34%	33%	21%	20%	21%
**History of hypertension**	7%	7%	7%	8%	6%	5%	17%	19%	19%
**History of hypercholesterolemia**	2%	3%	5%	15%	14%	15%	7%	9%	15%
**Total energy intake (kcal/d)**	2,159 (491)	1,746 (491)	1,407 (420)	2,238 (504)	1,777 (515)	1,489 (439)	2,444 (605)	1,969 (589)	1,686 (503)
**Saturated fat (percent of energy)**	14% (2.3)	13% (2.5)	11% (2.6)	13% (2.3)	11% (2.3)	9.4% (2.4)	13% (2.4)	11% (2.5)	8.4% (2.6)
**Polyunsaturated fat (percent of energy)**	6.4% (1.6)	6.6% (1.7)	6.9% (2.1)	5.5% (1.3)	5.7% (1.4)	5.8% (1.6)	5.6% (1.3)	5.9% (1.5)	6.2% (2.0)
**Monounsaturated fat (percent of energy)**	13% (2.0)	13% (2.3)	11% (2.7)	13% (2.1)	12% (2.3)	11% (2.6)	13% (2.1)	12% (2.5)	10% (3.1)
**Trans fat (percent of energy)**	2.1% (0.6)	2.0% (0.6)	1.6% (0.6)	1.9% (0.6)	1.7% (0.6)	1.2% (0.5)	1.6% (0.5)	1.3% (0.5)	0.8% (0.4)
**Cholesterol (mg/d)** ^**a**^	308 (86)	289 (96)	247 (105)	262 (63)	246 (66)	204 (73)	347 (110)	310 (108)	236 (102)
**Protein (percent of energy)**	16% (2.8)	18% (3.2)	19% (3.8)	18% (3.0)	19% (3.4)	20% (4.0)	17% (2.9)	19% (3.2)	19% (3.7)
**Carbohydrates (percent of energy)**	46% (6.9)	46% (7.8)	49% (8.8)	49% (6.7)	49% (7.3)	53% (8.5)	45% (6.8)	46% (8.1)	52% (10.1)
**Fiber (g/d)** ^**a**^	13 (2.7)	16 (3.8)	22 (5.9)	14 (2.9)	18 (4.0)	25 (6.9)	15 (3.7)	20 (5.2)	30 (8.7)
**Dietary folate (mcg/d)** ^**a**^	313 (174)	368 (211)	489 (290)	391 (241)	468 (289)	583 (328)	389 (202)	461 (253)	608 (354)
**Glycemic load** ^**a**^	100 (17)	99 (19)	102 (23)	120 (20)	120 (21)	127 (24)	122 (22)	122 (25)	135 (32)
**Glycemic index** ^**a**^	55 (2.8)	54 (3.5)	51 (4.2)	55 (2.8)	54 (3.2)	52 (3.6)	55 (3.0)	53 (3.4)	52 (4.2)
**Alcohol intake (g/d)**	7.1 (12)	7.2 (11)	6.4 (11)	2.8 (6.0)	3.0 (6.1)	3.4 (6.1)	12 (16)	12 (16)	11 (14)
**Food group intake (servings/day)** ^**a**^									
Whole grains	0.3 (0.7)	1.0 (1.0)	1.8 (1.3)	0.6 (0.8)	1.4 (1.0)	2.3 (1.3)	0.6 (0.9)	1.5 (1.2)	2.6 (1.7)
Fruits	0.6 (0.7)	1.2 (0.9)	2.2 (1.2)	0.5 (0.6)	1.2 (0.8)	1.9 (1.1)	0.8 (0.8)	1.5 (1.0)	2.7 (1.7)
Vegetables	1.9 (1.2)	2.9 (1.5)	4.5 (2.2)	1.8 (1.3)	3.2 (1.7)	5.0 (2.4)	1.9 (1.2)	3.0 (1.5)	4.8 (2.5)
Nuts	0.1 (0.4)	0.3 (0.4)	0.5 (0.5)	0.1 (0.3)	0.3 (0.3)	0.4 (0.4)	0.2 (0.5)	0.5 (0.6)	0.7 (0.8)
Legumes	0.2 (0.2)	0.4 (0.2)	0.5 (0.4)	0.2 (0.2)	0.4 (0.3)	0.6 (0.4)	0.3 (0.3)	0.4 (0.3)	0.7 (0.5)
Vegetable oil	0.2 (0.5)	0.5 (0.7)	1.0 (1.0)	0.1 (0.3)	0.3 (0.4)	0.5 (0.5)	0.1 (0.2)	0.2 (0.3)	0.4 (0.5)
Tea and coffee	2.4 (1.8)	3.0 (1.9)	3.6 (2.0)	1.5 (1.7)	2.2 (1.9)	2.8 (2.0)	1.8 (1.8)	2.4 (1.9)	2.6 (2.0)
Fruit juices	0.8 (0.8)	0.7 (0.7)	0.5 (0.6)	0.7 (0.9)	0.7 (0.8)	0.6 (0.7)	0.8 (0.9)	0.8 (0.9)	0.7 (0.8)
Refined grains	2.0 (1.5)	1.6 (1.3)	1.1 (0.9)	1.9 (1.2)	1.6 (0.9)	1.4 (0.7)	2.0 (1.3)	1.5 (1.1)	1.2 (0.8)
Potatoes	0.6 (0.4)	0.5 (0.3)	0.3 (0.2)	0.7 (0.4)	0.5 (0.3)	0.4 (0.2)	0.7 (0.4)	0.6 (0.4)	0.4 (0.3)
Sugar-sweetened beverages	0.5 (0.8)	0.2 (0.5)	0.1 (0.3)	0.9 (1.2)	0.5 (0.8)	0.2 (0.4)	0.7 (0.8)	0.3 (0.6)	0.2 (0.3)
Sweets and desserts	1.4 (1.3)	1.1 (1.1)	0.9 (0.7)	1.5 (1.3)	1.3 (1.0)	1.0 (0.7)	1.8 (1.5)	1.5 (1.2)	1.0 (0.9)
Animal fat	0.7 (1.1)	0.3 (0.7)	0.2 (0.4)	0.4 (0.7)	0.1 (0.4)	0.1 (0.2)	0.6 (1.0)	0.3 (0.6)	0.1 (0.3)
Dairy	1.8 (1.4)	1.8 (1.2)	1.8 (1.0)	2.3 (1.5)	2.3 (1.3)	2.2 (1.1)	2.2 (1.6)	2.0 (1.2)	1.7 (1.0)
Eggs	0.4 (0.4)	0.3 (0.3)	0.3 (0.3)	0.3 (0.2)	0.2 (0.2)	0.1 (0.1)	0.5 (0.5)	0.3 (0.4)	0.2 (0.3)
Fish and seafood	0.3 (0.2)	0.3 (0.2)	0.3 (0.3)	0.2 (0.2)	0.3 (0.2)	0.3 (0.3)	0.3 (0.3)	0.4 (0.3)	0.4 (0.4)
Poultry	0.2 (0.2)	0.3 (0.2)	0.3 (0.3)	0.3 (1.0)	0.3 (1.0)	0.4 (1.0)	0.8 (1.9)	0.9 (2.0)	1.0 (2.2)
Unprocessed red meat	0.7 (0.4)	0.6 (0.4)	0.5 (0.3)	0.4 (0.9)	0.4 (1.0)	0.3 (1.0)	0.7 (1.4)	0.6 (1.4)	0.5 (1.4)
Processed red meat	0.4 (0.4)	0.3 (0.3)	0.2 (0.2)	0.2 (0.5)	0.1 (0.6)	0.1 (0.5)	0.5 (1.3)	0.4 (1.4)	0.2 (1.1)
Miscellaneous animal-based foods	0.5 (0.4)	0.4 (0.4)	0.3 (0.3)	0.3 (0.8)	0.2 (0.7)	0.1 (0.5)	0.5 (1.6)	0.4 (1.3)	0.2 (0.9)

Data are mean (SD) for continuous variables and percentage for dichotomous variables, unless otherwise indicated.

^a^Values are energy-adjusted.

MET, metabolic equivalent of task.

### Plant-Based Diet Indices and Type 2 Diabetes Incidence

During 4,102,369 person-years of follow-up, we documented 16,162 T2D cases. PDI was inversely associated with T2D incidence in all three cohorts after adjusting for potential confounders (Table 2). Adjustment for BMI attenuated the relationship, but associations remained significant (pooled hazard ratio [HR] for extreme deciles 0.80, 95% CI 0.74–0.87; HR per 10-unit increase 0.88, 95% CI 0.86–0.91, *p* trend < 0.001).

**Table 2:** Hazard ratios (95% CIs) for type 2 diabetes according to deciles of the overall plant-based diet Index.

Cohort and Model	Decile 1	Decile 2	Decile 3	Decile 4	Decile 5	Decile 6	Decile 7	Decile 8	Decile 9	Decile 10	HR (95% CI) per 10 units	*p* Trend^a^
**NHS**												
Median	45.5	48.8	50.8	52.4	54.0	55.2	56.7	58.2	60.2	63.6		
Cases/person-years	902/165,059	901/162,584	839/168,132	883/165,825	776/164,319	729/167,845	750/169,967	640/159,687	686/175,345	605/163,941		
Age adjusted	1.00	1.00 (0.91, 1.09)	0.90 (0.82, 0.99)	0.96 (0.87, 1.05)	0.86 (0.78, 0.95)	0.79 (0.71, 0.87)	0.79 (0.72, 0.87)	0.72 (0.65, 0.79)	0.71 (0.64, 0.79)	0.66 (0.59, 0.73)	0.78 (0.75, 0.81)	<0.001
Multivariable adjusted	1.00	0.96 (0.87, 1.05)	0.85 (0.77, 0.93)	0.87 (0.79, 0.95)	0.77 (0.70, 0.85)	0.69 (0.63, 0.77)	0.68 (0.61, 0.75)	0.60 (0.54, 0.67)	0.59 (0.53, 0.66)	0.51 (0.46, 0.57)	0.68 (0.65, 0.72)	<0.001
Multivariable adjusted + BMI	1.00	1.00 (0.91, 1.10)	0.93 (0.85, 1.03)	0.99 (0.90, 1.09)	0.92 (0.83, 1.02)	0.87 (0.78, 0.96)	0.88 (0.80, 0.98)	0.81 (0.73, 0.90)	0.85 (0.76, 0.94)	0.83 (0.74, 0.93)	0.88 (0.84, 0.93)	<0.001
**NHS2**												
Median	45.3	48.8	51.0	52.5	54.0	55.3	57.0	58.7	61.0	64.3		
Cases/person-years	692/162,514	640/168,175	542/164,772	487/168,383	531/149,724	503/171,201	533/179,002	450/162,962	446/165,312	376/164,951		
Age adjusted	1.00	0.96 (0.83, 1.10)	0.82 (0.71, 0.94)	0.81 (0.70, 0.94)	0.81 (0.70, 0.93)	0.74 (0.63, 0.85)	0.72 (0.62, 0.83)	0.67 (0.58, 0.78)	0.69 (0.60, 0.80)	0.57 (0.48, 0.66)	0.77 (0.72, 0.81)	<0.001
Multivariable adjusted	1.00	0.94 (0.82, 1.08)	0.81 (0.70, 0.94)	0.80 (0.69, 0.93)	0.80 (0.69, 0.92)	0.72 (0.61, 0.83)	0.69 (0.60, 0.81)	0.64 (0.55, 0.75)	0.64 (0.55, 0.75)	0.53 (0.44, 0.62)	0.74 (0.69, 0.78)	<0.001
Multivariable adjusted + BMI	1.00	0.98 (0.88, 1.09)	0.88 (0.78, 0.98)	0.82 (0.73, 0.92)	0.94 (0.84, 1.06)	0.88 (0.78, 0.99)	0.97 (0.86, 1.09)	0.86 (0.75, 0.97)	0.91 (0.80, 1.03)	0.83 (0.72, 0.95)	0.93 (0.87, 0.98)	0.01
**HPFS**												
Median	45.0	48.5	50.6	52.3	54.0	55.5	57.0	58.6	61.0	64.4		
Cases/person-years	423/78,216	381/74,195	358/76,914	368/81,339	329/80,419	302/80,686	284/69,591	279/80,963	302/80,753	225/79,592		
Age adjusted	1.00	0.92 (0.80, 1.06)	0.83 (0.72, 0.95)	0.84 (0.73, 0.96)	0.75 (0.65, 0.87)	0.69 (0.60, 0.80)	0.70 (0.60, 0.82)	0.62 (0.53, 0.72)	0.68 (0.59, 0.79)	0.51 (0.43, 0.60)	0.73 (0.69, 0.77)	<0.001
Multivariable adjusted	1.00	0.90 (0.78, 1.03)	0.82 (0.71, 0.95)	0.82 (0.71, 0.94)	0.74 (0.64, 0.86)	0.68 (0.58, 0.79)	0.68 (0.58, 0.80)	0.59 (0.51, 0.70)	0.64 (0.54, 0.74)	0.48 (0.41, 0.57)	0.70 (0.66, 0.75)	<0.001
Multivariable adjusted + BMI	1.00	0.95 (0.83, 1.09)	0.92 (0.80, 1.06)	0.92 (0.80, 1.06)	0.87 (0.75, 1.00)	0.79 (0.68, 0.92)	0.84 (0.72, 0.98)	0.74 (0.63, 0.87)	0.85 (0.72, 0.99)	0.70 (0.59, 0.83)	0.84 (0.78, 0.89)	<0.001
**Pooled results (fixed-effects model)**												
Age adjusted	1.00	0.97 (0.91, 1.04)	0.86 (0.81, 0.92)	0.89 (0.84, 0.96)	0.82 (0.77, 0.88)	0.75 (0.70, 0.81)	0.75 (0.70, 0.81)	0.68 (0.63, 0.73)	0.70 (0.65, 0.75)	0.60^**b**^ (0.56, 0.65)	0.76 (0.74, 0.79)	<0.001
Multivariable adjusted	1.00	0.94 (0.88, 1.01)	0.83 (0.78, 0.89)	0.84 (0.78, 0.90)	0.77 (0.72, 0.83)	0.69 (0.64, 0.75)	0.68 (0.63, 0.73)	0.61 (0.56, 0.66)	0.61 (0.57, 0.66)	0.51 (0.47, 0.55)	0.70 (0.68, 0.73)	<0.001
Multivariable adjusted + BMI	1.00	0.99 (0.93, 1.05)	0.91 (0.85, 0.97)	0.92^b^ (0.86, 0.98)	0.92 (0.86, 0.98)	0.85 (0.80, 0.91)	0.90 (0.84, 0.97)	0.81 (0.75, 0.87)	0.87 (0.81, 0.93)	0.80 (0.74, 0.87)	0.88 (0.86, 0.91)	<0.001

Multivariable-adjusted model: adjusted for age (years), smoking status (never, past, current [1–14, 15–24, or ≥25 cigarettes/day]), physical activity (<3, 3–8.9, 9–17.9, 18–26.9, or ≥27 MET-h/wk), alcohol intake (0, 0.1–4.9, 5–9.9, 10–14.9, or ≥15 g/d), multivitamin use (yes or no), family history of diabetes (yes or no), margarine intake (quintiles), energy intake (quintiles), baseline hypertension (yes or no), baseline hypercholesterolemia (yes or no). Also adjusted for menopause status and postmenopausal hormone use in NHS and NHS2 (premenopausal or, if postmenopausal, current, past, or never postmenopausal hormone use) and for oral contraceptive use in NHS2 (never, past, or current use). Multivariable model + BMI: additionally adjusted for BMI (<21, 21–22.9, 23–24.9, 25–26.9, 27–29.9, 30–32.9, 33–34.9, 35–39.9, or ≥40 kg/m^2^).

^a^ *p*-Value when each decile was assigned the median value and treated as a continuous variable.

^b^ *p*-Value for *Q*-statistic < 0.05, indicating statistically significant heterogeneity among the three studies.

**Fig 1:**
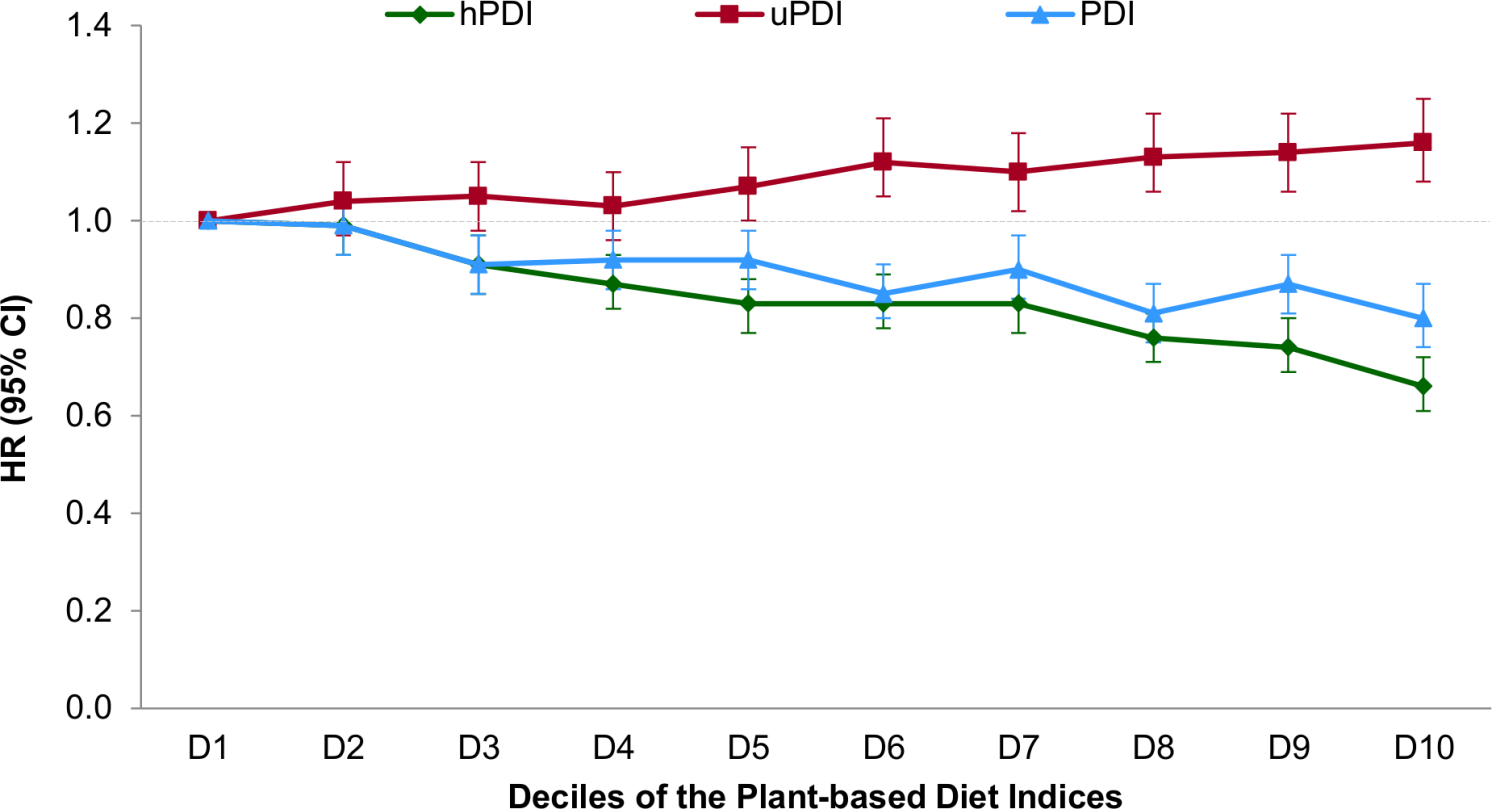
Pooled hazard ratios (95% CIs) for type 2 diabetes according to deciles of the overall, healthful, and unhealthful plant-based diet indices. Results were pooled across the three cohorts using a fixed-effects model. Adjusted for age (years), smoking status (never, past, current [1–14, 15–24, or ≥25 cigarettes/day]), physical activity (<3, 3–8.9, 9–17.9, 18–26.9, or ≥27 MET-h/wk), alcohol intake (0, 0.1–4.9, 5–9.9, 10–14.9, or ≥15 g/d), multivitamin use (yes or no), family history of diabetes (yes or no), margarine intake (quintiles), energy intake (quintiles), baseline hypertension (yes or no), baseline hypercholesterolemia (yes or no), and BMI (<21, 21–22.9, 23–24.9, 25–26.9, 27–29.9, 30–32.9, 33–34.9, 35–39.9, or ≥40 kg/m^2^). Also adjusted for menopausal status and postmenopausal hormone use in NHS and NHS2 (premenopausal or, if postmenopausal, current, past, or never postmenopausal hormone use) and for oral contraceptive use in NHS2 (never, past, or current use). *p* trend < 0.001 for all indices. *p*-Value obtained by assigning the median value to each decile and entering this as a continuous variable in the model.

After multivariable adjustment, a strong inverse association was observed between hPDI and T2D (Table 3), which was only modestly attenuated after BMI adjustment (pooled HR for extreme deciles 0.66, 95% CI 0.61–0.72; HR per 10-unit increase 0.83, 95% CI 0.80–0.85, *p* trend < 0.001). There was significant heterogeneity in the pooled estimates controlled for BMI due to greater attenuation in NHS2. In contrast, uPDI was positively associated with T2D (pooled HR for extreme deciles 1.16, 95% CI 1.08–1.25, *p* trend < 0.001) (Fig 1).

**Table 3:** Hazard ratios (95% CI) for type 2 diabetes according to deciles of the healthful plant-based diet index.

Cohort and Model	Decile 1	Decile 2	Decile 3	Decile 4	Decile 5	Decile 6	Decile 7	Decile 8	Decile 9	Decile 10	HR (95% CI) per 10 units	*p* Trend^a^
**NHS**												
Median	44.3	48.2	50.6	52.5	54.0	55.8	57.5	59.3	61.6	65.5		
Cases/person-years	1,054/165,958	993/168,094	871/168,590	805/168,233	705/158,011	783/170,962	748/165,507	654/162,229	615/168,844	483/166,277		
Age adjusted	1.00	0.91 (0.83, 0.99)	0.79 (0.72, 0.87)	0.73 (0.66, 0.80)	0.67 (0.61, 0.74)	0.68 (0.62, 0.75)	0.67 (0.61, 0.73)	0.59 (0.53, 0.65)	0.54 (0.49, 0.59)	0.42 (0.37, 0.47)	0.68 (0.66, 0.71)	<0.001
Multivariable adjusted	1.00	0.96 (0.88, 1.05)	0.85 (0.78, 0.93)	0.80 (0.73, 0.88)	0.75 (0.68, 0.83)	0.77 (0.70, 0.84)	0.76 (0.69, 0.84)	0.69 (0.62, 0.76)	0.65 (0.58, 0.72)	0.52 (0.46, 0.58)	0.75 (0.72, 0.78)	<0.001
Multivariable adjusted + BMI	1.00	0.98 (0.89, 1.06)	0.87 (0.79, 0.95)	0.82 (0.75, 0.90)	0.77 (0.70, 0.85)	0.79 (0.72, 0.87)	0.80 (0.72, 0.88)	0.73 (0.65, 0.80)	0.70 (0.63, 0.78)	0.60 (0.54, 0.68)	0.80 (0.76, 0.83)	<0.001
**NHS2**												
Median	44.0	48.0	50.3	52.3	54.0	55.8	57.4	59.2	61.7	66.0		
Cases/person-years	725/167,601	622/155,811	619/184,677	553/157,937	547/174,667	494/160,349	497/167,624	419/157,065	416/167,334	308/163,931		
Age adjusted	1.00	0.88 (0.79, 0.97)	0.77 (0.69, 0.86)	0.75 (0.67, 0.83)	0.67 (0.60, 0.75)	0.65 (0.58, 0.72)	0.61 (0.54, 0.68)	0.52 (0.46, 0.58)	0.49 (0.43, 0.55)	0.36 (0.31, 0.41)	0.64 (0.62, 0.67)	<0.001
Multivariable adjusted	1.00	0.99 (0.89, 1.10)	0.93 (0.84, 1.04)	0.93 (0.84, 1.04)	0.84 (0.75, 0.94)	0.85 (0.75, 0.95)	0.82 (0.73, 0.93)	0.73 (0.64, 0.83)	0.72 (0.64, 0.82)	0.58 (0.50, 0.66)	0.79 (0.75, 0.83)	<0.001
Multivariable adjusted + BMI	1.00	1.05 (0.94, 1.17)	0.99 (0.88, 1.10)	1.00 (0.89, 1.12)	0.92 (0.82, 1.03)	0.93 (0.82, 1.04)	0.93 (0.83, 1.05)	0.85 (0.75, 0.96)	0.86 (0.76, 0.98)	0.77 (0.67, 0.89)	0.89 (0.84, 0.93)	<0.001
**HPFS**												
Median	43.3	47.3	50.0	52.0	53.8	55.3	57.0	59.2	61.8	66.0		
Cases/person-years	397/78,048	366/76,324	368/82,538	359/80,966	346/77,813	307/70,470	328/86,144	275/73,681	268/79,989	237/76,697		
Age adjusted	1.00	0.92 (0.80, 1.06)	0.88 (0.76, 1.01)	0.82 (0.71, 0.95)	0.82 (0.71, 0.95)	0.76 (0.66, 0.88)	0.71 (0.61, 0.82)	0.66 (0.56, 0.77)	0.60 (0.51, 0.70)	0.54 (0.45, 0.63)	0.76 (0.72, 0.80)	<0.001
Multivariable adjusted	1.00	0.93 (0.81, 1.07)	0.89 (0.77, 1.02)	0.83 (0.71, 0.95)	0.83 (0.72, 0.96)	0.77 (0.66, 0.90)	0.73 (0.62, 0.85)	0.67 (0.57, 0.79)	0.63 (0.53, 0.74)	0.58 (0.49, 0.68)	0.78 (0.73, 0.82)	<0.001
Multivariable adjusted + BMI	1.00	0.93 (0.81, 1.07)	0.87 (0.76, 1.01)	0.81 (0.70, 0.94)	0.82 (0.70, 0.95)	0.78 (0.67, 0.91)	0.75 (0.64, 0.87)	0.70 (0.59, 0.82)	0.66 (0.56, 0.77)	0.65 (0.55, 0.77)	0.81 (0.77, 0.86)	<0.001
**Pooled results (fixed-effects model)**												
Age adjusted	1.00	0.90 (0.85, 0.96)	0.80 (0.75, 0.85)	0.75 (0.71, 0.80)	0.70^b^ (0.65, 0.74)	0.68 (0.64, 0.73)	0.66 (0.61, 0.70)	0.58 (0.54, 0.62)	0.53 (0.50, 0.57)	0.42^b^ (0.39, 0.45)	0.69^b^ (0.67, 0.70)	<0.001^**b**^
Multivariable adjusted	1.00	0.96 (0.91, 1.03)	0.88 (0.83, 0.94)	0.85 (0.79, 0.90)	0.80 (0.74, 0.85)	0.79 (0.74, 0.85)	0.77 (0.72, 0.83)	0.70 (0.65, 0.75)	0.67 (0.62, 0.72)	0.55 (0.51, 0.59)	0.77 (0.75, 0.79)	<0.001
Multivariable adjusted + BMI	1.00	0.99 (0.93, 1.05)	0.91 (0.85, 0.97)	0.87^b^ (0.82, 0.93)	0.83 (0.77, 0.88)	0.83 (0.78, 0.89)	0.83^b^ (0.77, 0.89)	0.76 (0.71, 0.81)	0.74^b^ (0.69, 0.80)	0.66^b^ (0.61, 0.72)	0.83^b^ (0.80, 0.85)	<0.001^**b**^

Multivariable-adjusted model: adjusted for age (years), smoking status (never, past, current [1–14, 15–24, or ≥25 cigarettes/day]), physical activity (<3, 3–8.9, 9–17.9, 18–26.9, or ≥27 MET-h/wk), alcohol intake (0, 0.1–4.9, 5–9.9, 10–14.9, or ≥15 g/d), multivitamin use (yes or no), family history of diabetes (yes or no), margarine intake (quintiles), energy intake (quintiles), baseline hypertension (yes or no), baseline hypercholesterolemia (yes or no). Also adjusted for menopause status and postmenopausal hormone use in NHS and NHS2 (premenopausal or, if postmenopausal, current, past, or never postmenopausal hormone use) and for oral contraceptive use in NHS2 (never, past, or current use). Multivariable model + BMI: additionally adjusted for BMI (<21, 21–22.9, 23–24.9, 25–26.9, 27–29.9, 30–32.9, 33–34.9, 35–39.9, or ≥40 kg/m^2^).

^a^ *p*-Value when each decile was assigned the median value and treated as a continuous variable. ^b^ *p*-Value for *Q*-statistic < 0.05, indicating statistically significant heterogeneity among the three studies.

### Sensitivity Analyses

**Fig 2:**
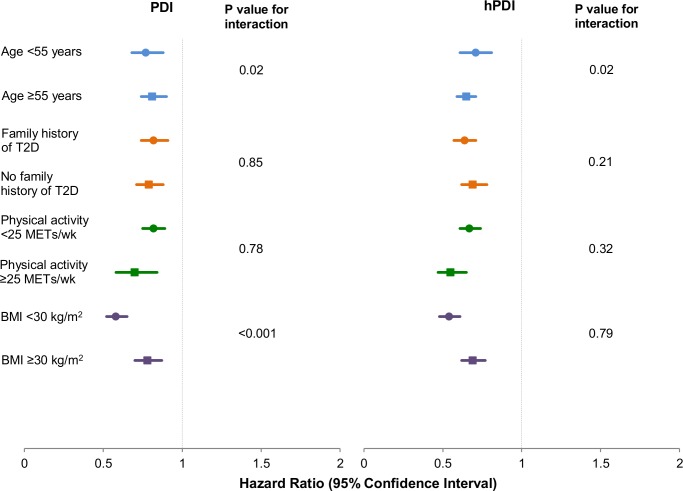
Pooled hazard ratios (95% CI) for type 2 diabetes comparing extreme deciles of the plant-based diet indices, stratified by selected characteristics. Results were pooled across the three cohorts using a fixed-effects model. Adjusted for age (years), smoking status (never, past, current [1–14, 15–24, or ≥25 cigarettes/day]), physical activity (<3, 3–8.9, 9–17.9, 18–26.9, or ≥27 MET-h/wk), alcohol intake (0, 0.1–4.9, 5–9.9, 10–14.9, or ≥15 g/d), multivitamin use (yes or no), family history of diabetes (yes or no), margarine intake (quintiles), energy intake (quintiles), baseline hypertension (yes or no), baseline hypercholesterolemia (yes or no), and BMI (<21, 21–22.9, 23–24.9, 25–26.9, 27–29.9, 30–32.9, 33–34.9, 35–39.9, or ≥40 kg/m^2^). Also adjusted for menopause status and postmenopausal hormone use in NHS and NHS2 (premenopausal or, if postmenopausal, current, past, or never postmenopausal hormone use) and for oral contraceptive use in NHS2 (never, past, or current use). *p* trend < 0.001 for both indices across all strata. *p*-Value obtained by assigning the median value to each decile and entering this as a continuous variable in the model.

Our findings remained robust in several sensitivity analyses. In restricted cubic spline analysis, we did not find evidence for a nonlinear association of either PDI or hPDI with T2D incidence. Thus, both indices had significant linear associations with T2D incidence, with a stronger dose-response relationship for hPDI (S1 Fig). Similar inverse associations were observed in strata defined by physical activity and family history of diabetes (Fig 2). The inverse association of PDI with T2D incidence was stronger in non-obese than in obese participants (*p* interaction < 0.001), and the inverse associations of both PDI and hPDI were stronger in older participants (*p* interaction = 0.02) (S3 Table). The associations of both PDI and hPDI with T2D were virtually unchanged upon further adjustment for ethnicity, marital status, recent physical exam, diet beverage intake, and indicators of socioeconomic status (S4 Table). Results were also similar when the analysis was restricted to participants with fasting plasma glucose screening in the previous 2 y (PDI: HR for extreme deciles 0.78, 95% CI 0.71–0.85, *p* trend < 0.001; hPDI: HR for extreme deciles 0.65, 95% CI 0.59–0.71, *p* trend < 0.001). Continuously updating PDI and hPDI throughout follow-up did not change results (S5 Table). When we used baseline intakes of PDI and hPDI, associations were modestly attenuated but remained significant (PDI: HR for extreme deciles 0.86, 95% CI 0.80–0.93, *p* trend < 0.001; hPDI: HR for extreme deciles 0.70, 95% CI 0.64–0.75, *p* trend < 0.001). Associations were also modestly attenuated when we used the most recent scores prior to diagnosis of T2D (PDI: HR for extreme deciles 0.84, 95% CI 0.78–0.91, *p* trend < 0.001; hPDI: HR for extreme deciles 0.74, 95% CI 0.69–0.80, *p* trend < 0.001). Stratified analysis showed no significant effect modification by ethnicity for the diet indices (*p* interaction was 0.92 for PDI, 0.14 for hPDI, and 0.94 for uPDI; S2 Fig).

To examine the individual contributions of healthy plant foods, less healthy plant foods, and animal foods to T2D risk, we included variables for all three food types simultaneously in the fully adjusted model; this allowed for mutual adjustment of the food types for one another, and hence an evaluation of their independent associations with T2D incidence. Healthy plant foods were inversely associated with T2D, while animal foods were positively associated, and less healthy plant foods were not associated, with risk (S3 Fig).

To examine the effect of consuming a healthful plant-based diet that is also high in intake of some animal foods known to be associated with reduced risk of several health outcomes (e.g., fish and yogurt [30–33]), we created two variations of hPDI. When we modified hPDI to score fish/seafood intake positively, the pooled HRs were slightly attenuated (HR for extreme deciles 0.73, 95% CI 0.68–0.79; HR per 10-unit increase 0.87, 95% CI 0.85–0.89, *p* trend < 0.001). Results for a modified hPDI with yogurt scored positively were not substantially different (HR for extreme deciles 0.65, 95% CI 0.60–0.71; HR per 10-unit increase 0.83, 95% CI 0.81–0.85, *p* trend < 0.001).

Previous analyses in these cohorts have found other dietary patterns such as the Mediterranean diet, the Alternate Healthy Eating Index (aHEI), and Dietary Approaches to Stop Hypertension (DASH) to be inversely associated with T2D [34–36]. Thus, in order to examine the independent associations of PDI and hPDI with T2D incidence, we individually controlled for these patterns (S6 and S7 Tables). Pooled HRs for both PDI and hPDI remained largely unchanged when the Mediterranean diet was controlled for, and were only slightly attenuated with aHEI or DASH in the same model.

## Discussion

We found significant linear inverse associations of plant-based diets, especially a healthier version (captured by hPDI), with T2D incidence in three prospective cohorts in the US. In contrast, a less healthy version of a plant-based diet (captured by uPDI) was associated with increased T2D risk. These associations were independent of BMI and other diabetes risk factors.

There are several mechanisms through which a healthful plant-based diet could lower the risk of T2D [37,38]. Such a diet would be rich in dietary fiber, antioxidants, unsaturated fatty acids, and micronutrients such as magnesium, and low in saturated fat. Randomized clinical trials have shown beneficial effects of diets high in viscous and soluble fiber on improving postprandial glucose as well as long-term glucose metabolism [39]. In addition, several prospective studies have shown dietary fiber to be associated with reduced levels of inflammatory markers [40,41]. Animal studies and epidemiologic studies among humans have shown antioxidants such as polyphenols to have beneficial effects on glucose metabolism, probably through reduced oxidative stress and improved endothelial function [42]. High unsaturated fatty acid and low saturated fat contents in diets have also been shown to have anti-inflammatory properties [43], while specific micronutrients such as magnesium are known to play a key role in glucose metabolism [44]. Thus, a healthful plant-based diet could enhance glycemic control, improve insulin sensitivity, and decrease chronic inflammation, thereby reducing T2D risk. In addition, the high fiber and low calorie contents of many plant foods could further reduce T2D risk by promoting weight loss/maintenance [37,38]. Another less well understood mechanism could be through the gut microbiome. A healthful plant-based diet could promote a gut microbial environment that facilitates the metabolism of fiber and polyphenols and discourages the metabolism of bile acids, choline and L-carnitine, and amino acids, further reducing T2D risk [45]. An unhealthful plant-based diet, on the other hand, would have high glycemic index and load, reduced fiber, lower micronutrient content, and higher calorie content, which could adversely affect the above-mentioned pathways, resulting in increased T2D risk [2,10,12]. Such a diet would also have a high level of added sugar, which has been shown to be strongly associated with increased weight gain and T2D risk [12,46]. Given that BMI represents a pathway through which plant-based diets may affect T2D risk, controlling for it would have resulted in an underestimation of these diets’ true effects. Results from the final model controlling for BMI characterize plant-based diet associations that are independent of their potential beneficial effects on body weight. The association of PDI with decreased T2D incidence was also significantly stronger for non-obese individuals than for obese individuals, which could represent a true biological interaction of PDI with BMI (e.g., due to differential mediation by BMI in obese and non-obese individuals) or could be a methodological artifact (e.g., as a result of differential confounding or measurement error in the two strata).

Only a few prospective studies have examined the association of plant-based diets with T2D. The Adventist Health Studies found significantly higher T2D mortality (odds ratio 1.9, 95% CI 1.2–3.1) and incidence (odds ratio 1.38, 95% CI 1.06–1.80) among non-vegetarians than vegetarians [7,8]. They also found consumption of vegan, lacto-ovo vegetarian, and semi-vegetarian diets to be associated with lower T2D risk relative to non-vegetarian diets [9]. All of these studies were carried out among Seventh-day Adventists, a religious group that encourages a lacto-ovo vegetarian diet. Because the prevalence of vegetarianism is low in the US (~3% [47]), it is difficult to study the relationship between vegetarianism and health outcomes in the general US population. Defining a plant-based diet in terms of a continuous gradation of adherence to a diet high in plant and low in animal foods has allowed us to study the association of plant-based diets with T2D in more than 200,000 participants, utilizing detailed dietary data collected at multiple time points over more than two decades.

Our study highlights the varying risk profiles associated with different versions of plant-based diets, emphasizing the importance of considering the quality of plant foods consumed. Participants in the highest decile of uPDI consumed half the amount of healthy plant foods and almost double the amount of less healthy plant foods consumed by participants in the highest decile of hPDI. The healthier version of a plant-based diet proposed in this study may inform future public health recommendations regarding plant-based diets. We also found that even a modest lowering in animal food consumption was associated with substantially lower T2D incidence. For instance, in the highest decile of hPDI, participants consumed ~4 servings/day of animal foods, relative to 5–6 servings/day in the lowest decile. This has important public health implications, as plant-based diets need not completely exclude animal foods. Numerous studies have previously documented null or inverse associations of several animal foods (e.g., low-fat dairy, lean poultry, and fish and seafood) with T2D and other diseases, and consistent positive associations of certain animal foods (e.g., red and processed meats) with such diseases. Additionally, in our analysis the association of hPDI with T2D changed only slightly upon positively scoring fish and yogurt intake. Thus, the gradual reduction in animal food intake suggested here can be achieved largely through reducing intake of low-quality animal foods.

Our findings provide support for the 2015 Dietary Guidelines Advisory Committee conclusion that diets rich in healthy plant foods and lower in certain animal foods such as red and processed meats are beneficial for the prevention of chronic diseases [6]. Another rationale for shifting towards a plant-based diet is to improve food sustainability because food systems that rely heavily on animal foods require more natural resources than those more reliant on plant foods [48]. Thus, dietary guidelines that recommend a healthful plant-based diet would be compatible with the health of humans as well as our ecosystem. The hPDI was only moderately correlated with other commonly considered dietary patterns such as the Mediterranean diet, aHEI, and DASH, reflecting that this is a novel diet index that captures unique aspects of a healthful plant-based diet. This, coupled with the strong inverse association of the hPDI with T2D independent of these other dietary patterns, highlights the importance of focusing on a healthful plant-based diet for a potentially environmentally sustainable approach to T2D prevention.

Our study has several limitations. Because diet was self-reported, measurement errors are inevitable. However, the use of cumulative measures of diet over time not only reduces these errors but also represents long-term dietary habits [18]. We also made assumptions about the healthfulness of different plant foods, which, although based on prior evidence, has an element of subjectivity, and hence our findings need to be replicated in future studies. While we controlled for several potential confounders, given the observational nature of these studies, residual or unmeasured confounding cannot be ruled out. However, several randomized controlled trials have found vegetarian diets to positively impact intermediate endpoints, such as body weight, blood pressure, lipid profile, and insulin sensitivity, in those who were free of T2D [49–51] and in patients with the disease [52–56]. The socioeconomic homogeneity of the study population also enhances internal validity due to implicit control of confounders. Given that we found similar associations between the plant-based diet indices and T2D among different ethnic groups, it is likely that these findings are generalizable to diverse racial/ethnic groups. Nevertheless, these studies were carried out among health professionals in the US, and hence it would be important to replicate these findings in other populations representing diverse countries and occupational groups before translating these findings to other populations.

### Conclusions

We found an inverse association between an overall plant-based diet and T2D incidence in three prospective cohorts. This inverse association was stronger for an index that captured a healthier version of the plant-based diet, but the association with T2D was positive for an index that captured an unhealthful version of a plant-based diet. Our study supports current recommendations to shift to diets rich in healthy plant foods, with lower intake of less healthy plant and animal foods.

## Supporting Information

S1 Fig Dose-response relationship between intake of plant-based diet indices and incidence of type 2 diabetes. Adjusted for age (years), smoking status (never, past, current [1–14, 15–24, or ≥25 cigarettes/day]), physical activity (<3, 3–8.9, 9–17.9, 18–26.9, or ≥27 MET-h/wk), alcohol intake (0, 0.1–4.9, 5–9.9, 10–14.9, or ≥15 g/d), multivitamin use (yes or no), family history of diabetes (yes or no), margarine intake (quintiles), energy intake (quintiles), baseline hypertension (yes or no), baseline hypercholesterolemia (yes or no), and BMI (<21, 21–22.9, 23–24.9, 25–26.9, 27–29.9, 30–32.9, 33–34.9, 35–39.9, or ≥40 kg/m^2^). Also adjusted for menopause status and postmenopausal hormone use in NHS and NHS2 (premenopausal or, if postmenopausal, current, past, or never postmenopausal hormone use) and oral contraceptive use in NHS2 (never, past, or current use). The graph is left-truncated, i.e., the *x*-axis begins at 35, as the minimum values of the cumulatively updated indices are 41.5 (PDI) and 40 (hPDI), and the value 0 is theoretically implausible. No spline variables got selected into the model based on stepwise selection; hence, the results of the model with the linear term alone have been shown for each index. Analysis carried out after combining all three cohorts.
(DOCX)

S2 Fig Hazard ratios (95% CI) for type 2 diabetes per 10-unit increase in adherence to plant-based diet indices, stratified by ethnicity. Adjusted for age, smoking status, physical activity, alcohol intake, multivitamin use, family history of diabetes, margarine intake, energy intake, baseline hypertension, baseline hypercholesterolemia, and BMI. Also adjusted for menopause status and postmenopausal hormone use in NHS and NHS2 and for oral contraceptive use in NHS2. *p* trend obtained by assigning the median value to each decile and entering this as a continuous variable in the model. *p* interaction between ethnicity and PDI = 0.92, between ethnicity and hPDI = 0.14, and between ethnicity and uPDI = 0.94. Analysis carried out after combining all three cohorts. *American Indian, Hawaiian, or other ancestry.
(DOCX)

S3 Fig Pooled hazard ratios (95% CIs) for type 2 diabetes according to deciles of animal, healthy plant, and less healthy plant foods (servings consumed/day). Adjusted for age, smoking status, physical activity, alcohol intake, multivitamin use, family history of diabetes, margarine intake, energy intake, baseline hypertension, baseline hypercholesterolemia, and BMI. Also adjusted for menopause status and postmenopausal hormone use in NHS and NHS2 and for oral contraceptive use in NHS2. Results were pooled across the three cohorts using a fixed-effects model. *p* trend = 0.49 for less healthy plant foods and <0.001 for healthy plant foods and animal foods. *p*-Value obtained by assigning the median value to each decile and entering this as a continuous variable in the model.
(DOCX)

S1 Table Examples of food items constituting the 18 food groups (from the 1984 NHS food frequency questionnaire). (DOCX)

S2 Table Age-standardized baseline characteristics by deciles of the overall plant-based diet index. (DOCX)

S3 Table Hazard ratios (95% CI) for type 2 diabetes according to deciles of the overall and healthful plant-based diet indices, stratified by age. (DOCX)

S4 Table Hazard ratios (95% CI) for type 2 diabetes according to deciles of the overall and healthful plant-based diet indices, controlling for additional variables. (DOCX)

S5 Table Pooled hazard ratios (95% CI) for type 2 diabetes according to deciles of the plant-based diet indices, with different ways of modeling diet. (DOCX)

S6 Table Pearson correlation coefficients between various dietary indices. (DOCX)

S7 Table Pooled hazard ratios (95% CI) for type 2 diabetes according to deciles of the overall and healthful plant-based diet indices, adjusting for other commonly used diet indices. (DOCX)

S1 Text STROBE statement. (DOCX)

S2 Text Prospective analysis plan. (DOCX)

## Abbreviations

aHEI: Alternate Healthy Eating Index
BMI: body mass index
CVD: cardiovascular disease
DASH: Dietary Approaches to Stop Hypertension
hPDI: healthful plant-based diet index
HPFS: Health Professionals Follow-Up Study
HR: hazard ratio
MET: metabolic equivalent of task
NHS: Nurses’ Health Study
NHS2: Nurses’ Health Study 2
PDI: overall plant-based diet index
T2D: type 2 diabetes
uPDI: unhealthful plant-based diet index
