# Supplementary material for: Plant-Based Dietary Patterns and Incidence of Type 2 Diabetes in US Men and Women: Results from Three Prospective Cohort Studies
Source: PLoS Med. 2016 Jun 14;13(6):e1002039. doi: 10.1371/journal.pmed.1002039 (PMC4907448; doi:10.1371/journal.pmed.1002039)
Supplement: S1 Fig — Adjusted for age (years), smoking status (never, past, current [1–14, 15–24, or ≥25 cigarettes/day]), physical activity (<3, 3–8.9, 9–17.9, 18–26.9, or ≥27 MET-h/wk), alcohol intake (0, 0.1–4.9, 5–9.9, 10–14.9, or ≥15 g/d), multivitamin use (yes or no), family history of diabetes (yes or no), margarine intake (quintiles), energy intake (quintiles), baseline hypertension (yes or no), baseline hypercholesterolemia (yes or no), and BMI (<21, 21–22.9, 23–24.9, 25–26.9, 27–29.9, 30–32.9, 33–34.9, 35–39.9, or ≥40 kg/m2). Also adjusted for menopause status and postmenopausal hormone use in NHS and NHS2 (premenopausal or, if postmenopausal, current, past, or never postmenopausal hormone use) and oral contraceptive use in NHS2 (never, past, or current use). The graph is left-truncated, i.e., the x-axis begins at 35, as the minimum values of the cumulatively updated indices are 41.5 (PDI) and 40 (hPDI), and the value 0 is theoretically implausible. No spline variables got selected into the model based on stepwise selection; hence, the results of the model with the linear term alone have been shown for each index. Analysis carried out after combining all three cohorts. (DOCX) [file pmed.1002039.s001.docx]

**S1 Fig. Dose-response relationship between intake of plant-based diet indices and incidence of type 2 diabetes**

**PDI**

P for linear trend <0.001

**hPDI**

P for linear trend <0.001

*Adjusted for age (years), smoking status (never, past, current [1-14, 15-24, or ≥25 cigarettes/day]), physical activity (<3, 3-8.9, 9-17.9, 18-26.9, or ≥27 MET-h/wk), alcohol intake (0, 0.1-4.9, 5-9.9, 10-14.9, or ≥15 g/day), multivitamin use (yes or no), family history of diabetes (yes or no), margarine intake, (quintiles), energy intake (quintiles), baseline hypertension (yes or no), baseline hypercholesterolemia (yes or no), and BMI (<21, 21-22.9, 23-24.9, 25-26.9, 27-29.9, 30-32.9, 33–34.9, 35-39.9, or ≥40 kg/m2). Also adjusted for menopause status and postmenopausal hormone use in NHS & NHS2 (premenopausal or, if postmenopausal, current, past, or never postmenopausal hormone use) and oral contraceptive use in NHS2 (never, past, or current use).*

*The graph is left-truncated, i.e., the x-axis begins at 35, as the minimum values of the cumulatively updated indices are 41.5 (PDI) and 40 (hPDI), and the value “0” is theoretically implausible*

*No spline variables got selected into the model based on stepwise selection; hence the results of the model with the linear term alone have been shown for each index*

*Analysis carried out after combining all three cohorts*
