# Supplementary material for: Plant-Based Dietary Patterns and Incidence of Type 2 Diabetes in US Men and Women: Results from Three Prospective Cohort Studies
Source: PLoS Med. 2016 Jun 14;13(6):e1002039. doi: 10.1371/journal.pmed.1002039 (PMC4907448; doi:10.1371/journal.pmed.1002039)
Supplement: S2 Fig — Adjusted for age, smoking status, physical activity, alcohol intake, multivitamin use, family history of diabetes, margarine intake, energy intake, baseline hypertension, baseline hypercholesterolemia, and BMI. Also adjusted for menopause status and postmenopausal hormone use in NHS and NHS2 and for oral contraceptive use in NHS2. p trend obtained by assigning the median value to each decile and entering this as a continuous variable in the model. p interaction between ethnicity and PDI = 0.92, between ethnicity and hPDI = 0.14, and between ethnicity and uPDI = 0.94. Analysis carried out after combining all three cohorts. *American Indian, Hawaiian, or other ancestry. (DOCX) [file pmed.1002039.s002.docx]

**S2 Fig. Hazard ratios (95% CI) for type 2 diabetes per 10-unit increase in adherence to plant-based diet indices, stratified by ethnicity**

*Adjusted for age, smoking status, physical activity, alcohol intake, multivitamin use, family history of diabetes, margarine intake, energy intake, baseline hypertension, baseline hypercholesterolemia, and BMI. Also adjusted for* *menopause status and postmenopausal hormone use in NHS & NHS2 and for oral contraceptive use in NHS2.*

*p-trend obtained by assigning the median value to each decile and entering this as a continuous variable in the model*

*p interaction between ethnicity and PDI=0.92, between ethnicity and hPDI=0.14, and between ethnicity and uPDI=0.94*

*Analysis carried out after combining all three cohorts*

**American Indian, Hawaiian, or other ancestry*
