# Supplementary material for: Plant-Based Dietary Patterns and Incidence of Type 2 Diabetes in US Men and Women: Results from Three Prospective Cohort Studies
Source: PLoS Med. 2016 Jun 14;13(6):e1002039. doi: 10.1371/journal.pmed.1002039 (PMC4907448; doi:10.1371/journal.pmed.1002039)
Supplement: S3 Fig — Adjusted for age, smoking status, physical activity, alcohol intake, multivitamin use, family history of diabetes, margarine intake, energy intake, baseline hypertension, baseline hypercholesterolemia, and BMI. Also adjusted for menopause status and postmenopausal hormone use in NHS and NHS2 and for oral contraceptive use in NHS2. Results were pooled across the three cohorts using a fixed-effects model. p trend = 0.49 for less healthy plant foods and <0.001 for healthy plant foods and animal foods. p-Value obtained by assigning the median value to each decile and entering this as a continuous variable in the model. (DOCX) [file pmed.1002039.s003.docx]

**S3 Fig. Pooled hazard ratios (95% CIs) for type 2 diabetes according to deciles of animal, healthy plant, and less healthy plant foods (servings consumed/day)**

*Adjusted for age, smoking status, physical activity, alcohol intake, multivitamin use, family history of diabetes, margarine intake, energy intake, baseline hypertension, baseline hypercholesterolemia, and BMI. Also adjusted for menopause status and postmenopausal hormone use in NHS & NHS2 and for oral contraceptive use in NHS2.*

*Results were pooled across the three cohorts using a fixed-effects model*

*p-trend=0.49 for less healthy plant foods, and <0.001 for healthy plant foods and animal foods. P-Value obtained by assigning the median value to each decile and entering this as a continuous variable in the model*
