# Supplementary material for: Plant-Based Dietary Patterns and Incidence of Type 2 Diabetes in US Men and Women: Results from Three Prospective Cohort Studies
Source: PLoS Med. 2016 Jun 14;13(6):e1002039. doi: 10.1371/journal.pmed.1002039 (PMC4907448; doi:10.1371/journal.pmed.1002039)
Supplement: S1 Table — (DOCX) [file pmed.1002039.s004.docx]

**S1 Table. Examples of food items constituting the 18 food groups (from the 1984 NHS food frequency questionnaire)**

| *Plant Food Groups* | |
| --- | --- |
| *Healthy* |  |
| Whole grains | Whole grain breakfast cereal, other cooked breakfast cereal, cooked oatmeal, dark bread, brown rice, other grains, bran, wheat germ, popcorn |
| Fruits | Raisins or grapes, prunes, bananas, cantaloupe, watermelon, fresh apples or pears, oranges, grapefruit, strawberries, blueberries, peaches or apricots or plums |
| Vegetables | Tomatoes, tomato juice, tomato sauce, broccoli, cabbage, cauliflower, Brussels sprouts, carrots, mixed vegetables, yellow or winter squash, eggplant or zucchini, yams or sweet potatoes, spinach cooked, spinach raw, kale or mustard or chard greens, iceberg or head lettuce, romaine or leaf lettuce, celery, mushrooms, beets, alfalfa sprouts, garlic, corn |
| Nuts | Nuts, peanut butter |
| Legumes | String beans, tofu or soybeans, beans or lentils, peas or lima beans |
| Vegetable oils | Oil-based salad dressing, vegetable oil used for cooking |
| Tea & Coffee | Tea, coffee, decaffeinated coffee |
| *Less healthy* |  |
| Fruit juices | Apple cider (non-alcoholic) or juice, orange juice, grapefruit juice, other fruit juice |
| Refined grains | Refined grain breakfast cereal, white bread, English muffins or bagels or rolls, muffins or biscuits, white rice, pancakes or waffles, crackers, pasta |
| Potatoes | French fries, baked or mashed potatoes, potato or corn chips |
| Sugar sweetened beverages | Colas with caffeine & sugar, colas without caffeine but with sugar, other carbonated beverages with sugar, non-carbonated fruit drinks with sugar |
| Sweets and Desserts | Chocolates, candy bars, candy without chocolate, cookies (home-baked & ready-made), brownies, doughnuts, cake (home-baked & ready-made), sweet roll (home-baked & ready-made), pie (home-baked & ready-made), jams or jellies or preserves or syrup or honey |
| *Animal Food Groups* | |
| Animal fat | Butter added to food, butter or lard used for cooking |
| Dairy | Skim low fat milk, whole milk, cream, sour cream, sherbet, ice cream, yogurt, cottage or ricotta cheese, cream cheese, other cheese |
| Egg | Eggs |
| Fish or Seafood | Canned tuna, dark meat fish, other fish, shrimp or lobster or scallops |
| Meat | Chicken or turkey with skin, chicken or turkey without skin, bacon, hot dogs, processed meats, liver, hamburger, beef or pork or lamb mixed dish, beef or pork or lamb main dish |
| Misc. animal-based foods | Pizza, chowder or cream soup, mayonnaise or other creamy salad dressing |
