# Supplementary material for: Plant-Based Dietary Patterns and Incidence of Type 2 Diabetes in US Men and Women: Results from Three Prospective Cohort Studies
Source: PLoS Med. 2016 Jun 14;13(6):e1002039. doi: 10.1371/journal.pmed.1002039 (PMC4907448; doi:10.1371/journal.pmed.1002039)
Supplement: S2 Table — (DOCX) [file pmed.1002039.s005.docx]

**S2 Table. Age-standardized baseline characteristics by deciles of the overall plant-based diet index**

|  | **NHS (1984)** | | | **NHS 2 (1991)** | | | **HPFS (1986)** | | |
| --- | --- | --- | --- | --- | --- | --- | --- | --- | --- |
| **Characteristic** | Decile 1 | Decile 5 | Decile 10 | Decile 1 | Decile 5 | Decile 10 | Decile 1 | Decile 5 | Decile 10 |
| Number of participants | 7750 | 8381 | 7270 | 9149 | 5382 | 8681 | 4567 | 4802 | 4424 |
| Median PDI | 44 | 54 | 65 | 44 | 54 | 66 | 44 | 54 | 65 |
| PDI range | 28-48 | 53-55 | 61-79 | 28-48 | 53-55 | 62-85 | 24-48 | 53-55 | 62-84 |
| Age (years) | 49 (6.9) | 50 (7.2) | 51 (7.3) | 36 (4.7) | 36 (4.7) | 37 (4.5) | 52 (9.4) | 53 (9.4) | 54 (9.5) |
| White | 98% | 98% | 98% | 96% | 97% | 97% | 95% | 95% | 95% |
| Current smoker | 31% | 24% | 19% | 17% | 12% | 9.6% | 14% | 9.5% | 5.3% |
| Physical activity (MET-h/wk) | 13 (21) | 14 (19) | 16 (23) | 17 (23) | 20 (26) | 27 (34) | 18 (26) | 20 (27) | 28 (34) |
| BMI (kg/m^2^) | 25 (5.0) | 25 (4.6) | 24 (4.2) | 25 (5.8) | 25 (5.2) | 24 (4.6) | 26 (3.4) | 25 (3.1) | 25 (3.0) |
| Current multivitamin use | 37% | 37% | 38% | 35% | 40% | 44% | 40% | 42% | 45% |
| Premenopausal | 51% | 49% | 41% | 97% | 97% | 96% | - | - | - |
| Current postmenopausal hormone use | 11% | 12% | 13% | 2.5% | 2.5% | 3.3% | - | - | - |
| Current oral contraceptive use | - | - | - | 13% | 11% | 8% | - | - | - |
| Family history of diabetes | 29% | 29% | 28% | 35% | 34% | 32% | 20% | 21% | 20% |
| History of hypertension | 9.4% | 8.0% | 6.9% | 6.8% | 6.6% | 5.2% | 20% | 19% | 19% |
| History of hypercholesterolemia | 2.9% | 3.1% | 4.3% | 15% | 15% | 15% | 7.8% | 9.2% | 15% |
| Total energy intake (kcal/d) | 1422 (444) | 1697 (481) | 2134 (518) | 1417 (447) | 1747 (500) | 2218 (517) | 1633 (508) | 1949 (580) | 2416 (620) |
| Saturated fat (percent of energy) | 14% (3.0) | 13% (2.4) | 11% (2.1) | 13% (2.6) | 11% (2.2) | 9.5% (2.1) | 13% (2.9) | 11% (2.5) | 9% (2.4) |
| Polyunsaturated fat (percent of energy) | 6.5% (2.0) | 6.6% (1.8) | 6.9% (1.6) | 5.7% (1.6) | 5.6% (1.3) | 5.6% (1.2) | 5.7% (1.6) | 5.9% (1.5) | 6.1% (1.5) |
| Monounsaturated fat (percent of energy) | 14% (2.7) | 13% (2.3) | 12% (2.2) | 13% (2.6) | 12% (2.4) | 11% (2.3) | 13.2% (2.7) | 12.4% (2.6) | 11.2% (2.7) |
| *Trans* fat (percent of energy) | 1.8% (0.6) | 1.9% (0.6) | 2.0% (0.6) | 1.8% (0.7) | 1.7% (0.6) | 1.5% (0.5) | 1.3% (0.5) | 1.3% (0.5) | 1.2% (0.5) |
| Cholesterol (mg/d)^a^ | 360 (127) | 285 (82) | 224 (65) | 300 (81) | 244 (61) | 189 (54) | 391 (137) | 310 (97) | 228 (78) |
| Protein (percent of energy) | 20% (4.0) | 18% (3.1) | 16% (2.4) | 22% (3.9) | 19% (3.2) | 17% (2.8) | 21% (3.9) | 19% (3.3) | 17% (2.8) |
| Carbohydrates (percent of energy) | 39% (8.2) | 46% (6.9) | 52% (6.3) | 43% (7.5) | 50% (6.5) | 56% (6.6) | 39% (8.1) | 47% (7.3) | 54% (7.6) |
| Fiber (g/d)^a^ | 13 (4.0) | 16 (4.4) | 20 (4.6) | 14 (4.2) | 18 (5.0) | 22 (5.8) | 16 (5.4) | 20 (5.9) | 26 (7.3) |
| Dietary Folate (mcg/d)^a^ | 360 (262) | 378 (230) | 404 (193) | 445 (323) | 479 (294) | 511 (251) | 431 (299) | 470 (268) | 531 (252) |
| Glycemic Load^a^ | 82 (20) | 99 (18) | 112 (16) | 105 (22) | 122 (20) | 135 (19) | 103 (25) | 123 (23) | 143 (24) |
| Glycemic Index^a^ | 52 (4.8) | 54 (3.7) | 54 (2.8) | 53 (4.2) | 54 (3.3) | 54 (2.7) | 52 (4.5) | 53 (3.5) | 54 (3.1) |
| Alcohol intake (g/d) | 9.3 (14) | 6.9 (11) | 5.7 (8.7) | 3.5 (7.6) | 2.9 (5.7) | 3.3 (5.7) | 14 (18) | 11 (15) | 9.6 (13) |
| Food group intake (servings/day)^a^ |  |  |  |  |  |  |  |  |  |
| Whole grains | 0.8 (0.9) | 1.0 (1.0) | 1.5 (1.2) | 1.1 (0.8) | 1.4 (1.1) | 2.0 (1.4) | 1.1 (0.9) | 1.4 (1.2) | 2.2 (1.7) |
| Fruits | 0.9 (0.8) | 1.3 (1.0) | 1.8 (1.2) | 0.9 (0.6) | 1.2 (0.9) | 1.7 (1.1) | 1.1 (0.8) | 1.5 (1.2) | 2.2 (1.5) |
| Vegetables | 2.6 (1.4) | 2.9 (1.7) | 3.7 (2.0) | 2.6 (1.4) | 3.1 (1.8) | 4.2 (2.3) | 2.5 (1.4) | 3.0 (1.6) | 4.2 (2.3) |
| Nuts | 0.2 (0.3) | 0.3 (0.4) | 0.4 (0.6) | 0.2 (0.2) | 0.2 (0.3) | 0.4 (0.5) | 0.3 (0.4) | 0.5 (0.6) | 0.7 (0.8) |
| Legumes | 0.3 (0.2) | 0.4 (0.3) | 0.5 (0.3) | 0.3 (0.2) | 0.4 (0.3) | 0.6 (0.5) | 0.3 (0.2) | 0.4 (0.3) | 0.6 (0.5) |
| Vegetable oil | 0.4 (0.5) | 0.5 (0.7) | 0.8 (0.9) | 0.2 (0.3) | 0.3 (0.4) | 0.4 (0.5) | 0.2 (0.3) | 0.2 (0.4) | 0.4 (0.5) |
| Tea & Coffee | 2.6 (1.9) | 3.0 (1.9) | 3.5 (2.0) | 1.8 (1.8) | 2.2 (1.9) | 2.7 (2.0) | 2.1 (1.8) | 2.4 (1.9) | 2.6 (2.0) |
| Fruit juices | 0.5 (0.6) | 0.7 (0.7) | 0.9 (0.9) | 0.5 (0.5) | 0.7 (0.7) | 1.0 (1.0) | 0.6 (0.7) | 0.7 (0.8) | 1.1 (1.0) |
| Refined grains | 1.3 (1.0) | 1.5 (1.2) | 1.7 (1.5) | 1.4 (0.8) | 1.6 (0.9) | 1.7 (1.1) | 1.3 (0.9) | 1.5 (1.1) | 1.7 (1.3) |
| Potatoes | 0.4 (0.3) | 0.5 (0.3) | 0.5 (0.4) | 0.5 (0.3) | 0.5 (0.3) | 0.6 (0.4) | 0.5 (0.3) | 0.6 (0.4) | 0.6 (0.5) |
| Sugar-sweetened beverages | 0.2 (0.5) | 0.3 (0.6) | 0.3 (0.6) | 0.5 (0.8) | 0.5 (0.8) | 0.4 (0.8) | 0.3 (0.5) | 0.4 (0.6) | 0.4 (0.6) |
| Sweets & desserts | 0.9 (0.8) | 1.1 (1.0) | 1.3 (1.3) | 1.1 (0.8) | 1.2 (1.0) | 1.4 (1.1) | 1.2 (0.9) | 1.4 (1.2) | 1.6 (1.5) |
| Animal Fat | 0.6 (0.9) | 0.4 (0.8) | 0.1 (0.6) | 0.3 (0.5) | 0.2 (0.4) | 0.0 (0.3) | 0.5 (0.7) | 0.3 (0.6) | 0.0 (0.4) |
| Dairy | 2.3 (1.3) | 1.9 (1.2) | 1.3 (1.1) | 2.8 (1.4) | 2.4 (1.2) | 1.7 (1.3) | 2.5 (1.4) | 2.0 (1.3) | 1.4 (1.2) |
| Eggs | 0.5 (0.4) | 0.3 (0.3) | 0.2 (0.2) | 0.3 (0.2) | 0.2 (0.2) | 0.1 (0.2) | 0.5 (0.5) | 0.4 (0.4) | 0.2 (0.3) |
| Fish & seafood | 0.4 (0.3) | 0.3 (0.3) | 0.3 (0.2) | 0.3 (0.2) | 0.3 (0.2) | 0.2 (0.3) | 0.4 (0.3) | 0.4 (0.4) | 0.4 (0.4) |
| Poultry | 0.3 (0.3) | 0.3 (0.2) | 0.3 (0.2) | 0.4 (1.1) | 0.3 (1.0) | 0.3 (1.1) | 0.9 (2.2) | 0.8 (2.0) | 0.9 (2.2) |
| Unprocessed red meat | 0.7 (0.4) | 0.6 (0.3) | 0.5 (0.4) | 0.4 (1.0) | 0.4 (1.0) | 0.3 (1.1) | 0.7 (1.5) | 0.6 (1.3) | 0.5 (1.3) |
| Processed red meat | 0.4 (0.4) | 0.3 (0.3) | 0.2 (0.3) | 0.1 (0.6) | 0.1 (0.5) | 0.1 (0.6) | 0.6 (1.7) | 0.4 (1.4) | 0.3 (1.2) |
| Miscellaneous animal-based foods | 0.5 (0.4) | 0.4 (0.4) | 0.3 (0.3) | 0.4 (1.0) | 0.2 (0.6) | 0.1 (0.5) | 0.7 (2.1) | 0.3 (1.3) | 0.2 (0.8) |

*Data are mean (SD) for continuous variables and percentage for dichotomous variables, unless otherwise indicated*

*^a^ Values are energy-adjusted*

*MET, metabolic equivalent task*
