# Supplementary material for: Plant-Based Dietary Patterns and Incidence of Type 2 Diabetes in US Men and Women: Results from Three Prospective Cohort Studies
Source: PLoS Med. 2016 Jun 14;13(6):e1002039. doi: 10.1371/journal.pmed.1002039 (PMC4907448; doi:10.1371/journal.pmed.1002039)
Supplement: S3 Table — (DOCX) [file pmed.1002039.s006.docx]

**S3 Table. Hazard ratios (95% CI) for type 2 diabetes according to deciles of the overall & healthful plant-based diet indices, stratified by age**

|  | Decile 1 | Decile 2 | Decile 3 | Decile 4 | Decile 5 | Decile 6 | Decile 7 | Decile 8 | Decile 9 | Decile 10 | *p*  Trend^a^ |
| --- | --- | --- | --- | --- | --- | --- | --- | --- | --- | --- | --- |
| **OVERALL PLANT-BASED DIET INDEX** | | | | | | | | | | | |
| **Nurses’ Health Study** | | | | | | | | | | | |
| <55 years | 1.00 | 1.09  (0.87, 1.36) | 0.99  (0.78, 1.24) | 0.95  (0.75, 1.20) | 0.93  (0.73, 1.19) | 0.79  (0.61, 1.03) | 0.93  (0.72, 1.20) | 0.81  (0.62, 1.07) | 0.92  (0.70, 1.21) | 0.73  (0.53, 1.01) | 0.02 |
| ≥55 years | 1.00 | 0.99  (0.89, 1.10) | 0.93  (0.84, 1.03) | 1.00  (0.90, 1.11) | 0.92  (0.83, 1.03) | 0.88  (0.79, 0.98) | 0.88  (0.78, 0.98) | 0.81  (0.72, 0.91) | 0.84  (0.74, 0.94) | 0.84  (0.74, 0.95) | <0.001 |
| **Nurses’ Health Study 2** | | | | | | | | | | | |
| <55 years | 1.00 | 1.01  (0.89, 1.13) | 0.89  (0.78, 1.00) | 0.82  (0.72, 0.93) | 0.94  (0.83, 1.07) | 0.87  (0.76, 0.99) | 0.98  (0.86, 1.12) | 0.82  (0.71, 0.94) | 0.95  (0.83, 1.10) | 0.80  (0.69, 0.93) | 0.01 |
| ≥55 years | 1.00 | 0.90  (0.70, 1.17) | 0.84  (0.64, 1.09) | 0.81  (0.62, 1.07) | 0.95  (0.73, 1.23) | 0.92  (0.71, 1.19) | 0.89  (0.67, 1.17) | 1.00  (0.76, 1.31) | 0.76  (0.56, 1.02) | 0.95  (0.71, 1.27) | 0.64 |
| **Health Professionals Follow-Up Study** | | | | | | | | | | | |
| <55 years | 1.00 | 1.24  (0.90, 1.71) | 0.87  (0.61, 1.24) | 0.92  (0.65, 1.30) | 0.88  (0.61, 1.28) | 0.80  (0.54, 1.17) | 0.93  (0.63, 1.39) | 0.85  (0.57, 1.26) | 0.77  (0.51, 1.17) | 0.63  (0.39, 1.01) | 0.01 |
| ≥55 years | 1.00 | 0.91  (0.78, 1.06) | 0.93  (0.79, 1.09) | 0.92  (0.79, 1.08) | 0.87  (0.74, 1.02) | 0.80  (0.67, 0.94) | 0.82  (0.69, 0.97) | 0.73  (0.61, 0.86) | 0.86  (0.73, 1.02) | 0.71  (0.59, 0.86) | <0.001 |
| **Pooled results (fixed-effects model)** | | | | | | | | | | | |
| <55 years | 1.00 | 1.04  (0.94, 1.15) | 0.90  (0.81, 1.01) | 0.85  (0.77, 0.95) | 0.93  (0.84, 1.04) | 0.85  (0.76, 0.95) | 0.97  (0.87, 1.08) | 0.82  (0.73, 0.93) | 0.93  (0.82, 1.05) | 0.77  (0.68, 0.88) | <0.001 |
| ≥55 years | 1.00 | 0.96  (0.88, 1.04) | 0.92  (0.85, 1.00) | 0.96  (0.88, 1.04) | 0.91  (0.84, 0.99) | 0.86  (0.79, 0.94) | 0.86  (0.79, 0.94) | 0.81  (0.74, 0.88) | 0.84  (0.76, 0.92) | 0.81  (0.74, 0.90) | <0.001 |
| **HEALTHFUL PLANT-BASED DIET INDEX** | | | | | | | | | | | |
| **Nurses’ Health Study** | | | | | | | | | | | |
| <55 years | 1.00 | 0.86  (0.70, 1.05) | 0.90  (0.73, 1.11) | 0.79  (0.63, 0.98) | 0.74  (0.58, 0.94) | 0.72  (0.57, 0.92) | 0.73  (0.56, 0.94) | 0.72  (0.55, 0.94) | 0.60  (0.44, 0.81) | 0.54  (0.37, 0.77) | <0.001 |
| ≥55 years | 1.00 | 1.01  (0.92, 1.11) | 0.87  (0.79, 0.96) | 0.83  (0.75, 0.92) | 0.78  (0.70, 0.87) | 0.81  (0.73, 0.90) | 0.81  (0.73, 0.91) | 0.73  (0.65, 0.82) | 0.72  (0.65, 0.81) | 0.62  (0.55, 0.70) | <0.001 |
| **Nurses’ Health Study 2** | | | | | | | | | | | |
| <55 years | 1.00 | 1.04  (0.93, 1.18) | 1.02  (0.91, 1.15) | 1.03  (0.91, 1.17) | 0.90  (0.79, 1.02) | 0.93  (0.82, 1.06) | 0.95  (0.83, 1.09) | 0.86  (0.74, 0.99) | 0.82  (0.71, 0.95) | 0.76  (0.65, 0.89) | <0.001 |
| ≥55 years | 1.00 | 1.07  (0.82, 1.38) | 0.83  (0.63, 1.09) | 0.86  (0.65, 1.13) | 0.99  (0.76, 1.29) | 0.90  (0.69, 1.18) | 0.87  (0.66, 1.14) | 0.81  (0.61, 1.07) | 0.97  (0.74, 1.27) | 0.78  (0.58, 1.06) | 0.12 |
| **Health Professionals Follow-Up Study** | | | | | | | | | | | |
| <55 years | 1.00 | 0.90  (0.65, 1.23) | 1.01  (0.74, 1.39) | 0.64  (0.45, 0.91) | 0.86  (0.60, 1.22) | 0.55  (0.36, 0.84) | 0.60  (0.40, 0.90) | 0.48  (0.30, 0.78) | 0.87  (0.59, 1.29) | 0.60  (0.37, 0.96) | 0.002 |
| ≥55 years | 1.00 | 0.95  (0.80, 1.11) | 0.86  (0.73, 1.01) | 0.85  (0.72, 1.00) | 0.82  (0.70, 0.97) | 0.83  (0.70, 0.98) | 0.78  (0.66, 0.92) | 0.74  (0.62, 0.88) | 0.63  (0.53, 0.76) | 0.66  (0.55, 0.80) | <0.001 |
| **Pooled results (fixed-effects model)** | | | | | | | | | | | |
| <55 years | 1.00 | 0.98  (0.89, 1.08) | 0.99  (0.90, 1.09) | 0.93^b^  (0.84, 1.04) | 0.86  (0.77, 0.96) | 0.85^b^  (0.76, 0.95) | 0.87^b^  (0.78, 0.97) | 0.80  (0.71, 0.90) | 0.78  (0.69, 0.88) | 0.71  (0.61, 0.81) | <0.001 |
| ≥55 years | 1.00 | 1.00  (0.92, 1.08) | 0.86  (0.80, 0.94) | 0.84  (0.77, 0.91) | 0.81  (0.74, 0.88) | 0.82  (0.76, 0.90) | 0.81  (0.74, 0.88) | 0.74  (0.68, 0.81) | 0.72^b^  (0.66, 0.79) | 0.65  (0.59, 0.71) | <0.001 |

*Adjusted for age, smoking status, physical activity, alcohol intake, multivitamin use, family history of diabetes, margarine intake, energy intake, baseline hypertension, baseline hypercholesterolemia, and BMI. Also adjusted for menopause status and postmenopausal hormone use in NHS & NHS2, and for oral contraceptive use in NHS2.*

*^a^ p-Value when we assigned the median value to each decile and entered this as a continuous variable in the model*

*^b^ p-Value for Q-statistic for heterogeneity <0.05, indicating statistically significant heterogeneity in HRs among the three studies*
