# Supplementary material for: Plant-Based Dietary Patterns and Incidence of Type 2 Diabetes in US Men and Women: Results from Three Prospective Cohort Studies
Source: PLoS Med. 2016 Jun 14;13(6):e1002039. doi: 10.1371/journal.pmed.1002039 (PMC4907448; doi:10.1371/journal.pmed.1002039)
Supplement: S4 Table — (DOCX) [file pmed.1002039.s007.docx]

**S4 Table. Hazard ratios (95% CI) for type 2 diabetes according to deciles of the overall ands healthful plant-based diet indices, controlling for additional variables**

| Decile 1 | Decile 2 | Decile 3 | Decile 4 | Decile 5 | Decile 6 | Decile 7 | Decile 8 | Decile 9 | Decile 10 | HR (95% CI) per 10 units | *p*  Trend^a^ |
| --- | --- | --- | --- | --- | --- | --- | --- | --- | --- | --- | --- |
| **OVERALL PLANT-BASED DIET INDEX** | | | | | | | | | | | |
| **Nurses’ Health Study** | | | | | | | | | | | |
| 1.00 | 1.01  (0.92, 1.10) | 0.94  (0.85, 1.03) | 1.00  (0.91, 1.10) | 0.93  (0.84, 1.02) | 0.87  (0.79, 0.97) | 0.89  (0.81, 0.99) | 0.82  (0.74, 0.91) | 0.85  (0.77, 0.95) | 0.84  (0.75, 0.94) | 0.89  (0.84, 0.93) | <0.001 |
| **Nurses’ Health Study 2** | | | | | | | | | | | |
| 1.00 | 0.99  (0.89, 1.10) | 0.88  (0.79, 0.99) | 0.83  (0.73, 0.93) | 0.95  (0.85, 1.07) | 0.88  (0.78, 1.00) | 0.98  (0.87, 1.11) | 0.87  (0.77, 0.99) | 0.93  (0.82, 1.06) | 0.84  (0.73, 0.97) | 0.94  (0.88, 0.99) | 0.04 |
| **Health Professionals Follow-Up Study** | | | | | | | | | | | |
| 1.00 | 0.95  (0.83, 1.10) | 0.92  (0.80, 1.06) | 0.93  (0.80, 1.07) | 0.87  (0.75, 1.01) | 0.80  (0.69, 0.93) | 0.84  (0.72, 0.98) | 0.75  (0.64, 0.87) | 0.85  (0.73, 1.00) | 0.70  (0.59, 0.83) | 0.84  (0.78, 0.90) | <0.001 |
| **Pooled results (fixed-effects model)** | | | | | | | | | | | |
| 1.00 | 0.99  (0.93, 1.05) | 0.92  (0.86, 0.98) | 0.93  (0.87, 0.99) | 0.92  (0.86, 0.99) | 0.86  (0.80, 0.92) | 0.91  (0.85, 0.98) | 0.82  (0.76, 0.88) | 0.88  (0.82, 0.94) | 0.81  (0.75, 0.88) | 0.89^b^  (0.86, 0.92) | <0.001 |
| **HEALTHFUL PLANT-BASED DIET INDEX** | | | | | | | | | | | |
| **Nurses’ Health Study** | | | | | | | | | | | |
| 1.00 | 0.98  (0.89, 1.06) | 0.87  (0.79, 0.95) | 0.82  (0.74, 0.90) | 0.76  (0.69, 0.84) | 0.79  (0.72, 0.87) | 0.79  (0.72, 0.88) | 0.72  (0.65, 0.80) | 0.70  (0.63, 0.78) | 0.60  (0.53, 0.67) | 0.79  (0.76, 0.83) | <0.001 |
| **Nurses’ Health Study 2** | | | | | | | | | | | |
| 1.00 | 1.04  (0.94, 1.16) | 0.98  (0.88, 1.10) | 1.00  (0.89, 1.12) | 0.91  (0.81, 1.02) | 0.92  (0.82, 1.04) | 0.93  (0.82, 1.05) | 0.84  (0.74, 0.96) | 0.85  (0.75, 0.97) | 0.76  (0.66, 0.88) | 0.88  (0.84, 0.93) | <0.001 |
| **Health Professionals Follow-Up Study** | | | | | | | | | | | |
| 1.00 | 0.93  (0.80, 1.07) | 0.87  (0.75, 1.00) | 0.80  (0.69, 0.93) | 0.80  (0.69, 0.93) | 0.77  (0.66, 0.90) | 0.74  (0.63, 0.86) | 0.69  (0.59, 0.82) | 0.65  (0.55, 0.77) | 0.64  (0.54, 0.76) | 0.81  (0.76, 0.86) | <0.001 |
| **Pooled results (fixed-effects model)** | | | | | | | | | | | |
| 1.00 | 0.99  (0.93, 1.05) | 0.90  (0.85, 0.96) | 0.87^b^  (0.81, 0.93) | 0.82  (0.77, 0.88) | 0.83  (0.77, 0.88) | 0.82^b^  (0.77, 0.88) | 0.75  (0.70, 0.81) | 0.73^b^  (0.68, 0.79) | 0.66^b^  (0.61, 0.71) | 0.82^b^  (0.80, 0.85) | <0.001^b^ |

*Adjusted for variables in the multivariable adjusted model + BMI, and ethnicity (White, Black, Other), marital status (married, widowed, divorced/separated, never married), getting a physical exam in the previous year (yes or no), and diet beverage intake (quintiles). Also adjusted for husband’s education (high school or less, undergraduate, graduate school) in NHS & NHS2, family income (<29000, 30000-39000, 40000-50000, 50000-74000, 75000-99000, 100000-149000, ≥150000) in NHS2, and work status (full-time, part-time, retired), and profession (dentist, pharmacist, optometrist, osteopath, podiatrist, vet) in HPFS*

*^a^ p-Value when we assigned the median value to each decile and entered this as a continuous variable in the model*

*^b^ p-Value for Q-statistic for heterogeneity <0.05, indicating statistically significant heterogeneity in HRs among the three studies*
