# Supplementary material for: Plant-Based Dietary Patterns and Incidence of Type 2 Diabetes in US Men and Women: Results from Three Prospective Cohort Studies
Source: PLoS Med. 2016 Jun 14;13(6):e1002039. doi: 10.1371/journal.pmed.1002039 (PMC4907448; doi:10.1371/journal.pmed.1002039)
Supplement: S6 Table — (DOCX) [file pmed.1002039.s009.docx]

**S6 Table. Pearson correlation coefficients between various dietary indices**

|  | PDI | hPDI | uPDI | aMED | aHEI | DASH |
| --- | --- | --- | --- | --- | --- | --- |
| **Nurses’ Health Study** | | | | | | |
| PDI | 1.00 | 0.21 | –0.11 | 0.52 | 0.12 | 0.37 |
| hPDI | 0.21 | 1.00 | –0.36 | 0.37 | 0.66 | 0.61 |
| uPDI | –0.11 | –0.36 | 1.00 | –0.62 | –0.54 | –0.60 |
| aMED | 0.52 | 0.37 | –0.62 | 1.00 | 0.63 | 0.74 |
| aHEI | 0.12 | 0.66 | –0.54 | 0.63 | 1.00 | 0.70 |
| DASH | 0.37 | 0.61 | –0.60 | 0.74 | 0.70 | 1.00 |
| **Nurses’ Health Study 2** | | | | | | |
| PDI | 1.00 | 0.26 | –0.21 | 0.59 | 0.20 | 0.44 |
| hPDI | 0.26 | 1.00 | –0.33 | 0.35 | 0.68 | 0.60 |
| uPDI | –0.21 | –0.33 | 1.00 | –0.63 | –0.56 | –0.58 |
| aMED | 0.59 | 0.35 | –0.63 | 1.00 | 0.61 | 0.73 |
| aHEI | 0.20 | 0.68 | –0.56 | 0.61 | 1.00 | 0.71 |
| DASH | 0.44 | 0.60 | –0.58 | 0.73 | 0.71 | 1.00 |
| **Health Professionals Follow-Up Study** | | | | | | |
| PDI | 1.00 | 0.32 | –0.10 | 0.58 | 0.26 | 0.47 |
| hPDI | 0.32 | 1.00 | –0.30 | 0.42 | 0.66 | 0.54 |
| uPDI | –0.10 | –0.30 | 1.00 | –0.53 | –0.47 | –0.52 |
| aMED | 0.58 | 0.42 | –0.53 | 1.00 | 0.67 | 0.75 |
| aHEI | 0.26 | 0.66 | –0.47 | 0.67 | 1.00 | 0.68 |
| DASH | 0.47 | 0.54 | –0.52 | 0.75 | 0.68 | 1.00 |

*p-Value <0.001 for all correlation coefficients*

*Abbreviations: PDI, Overall Plant-based Diet Index; hPDI, Healthful Plant-based Diet Index;*

*uPDI, Unhealthful Plant-based Diet Index; aMED, Alternate Mediterranean Dietary Pattern;*

*aHEI, Alternate Healthy Eating Index; DASH, Dietary Approaches to Stop Hypertension*
