# Supplementary material for: Plant-Based Dietary Patterns and Incidence of Type 2 Diabetes in US Men and Women: Results from Three Prospective Cohort Studies
Source: PLoS Med. 2016 Jun 14;13(6):e1002039. doi: 10.1371/journal.pmed.1002039 (PMC4907448; doi:10.1371/journal.pmed.1002039)
Supplement: S7 Table — (DOCX) [file pmed.1002039.s010.docx]

**S7 Table. Pooled hazard ratios (95% CI) for type 2 diabetes according to deciles of the overall and healthful plant-based diet indices, adjusting for other commonly used diet indices**

|  | Decile 1 | Decile 2 | Decile 3 | Decile 4 | Decile 5 | Decile 6 | Decile 7 | Decile 8 | Decile 9 | Decile 10 | HR (95% CI) per 10 units | *p*  Trend^a^ |
| --- | --- | --- | --- | --- | --- | --- | --- | --- | --- | --- | --- | --- |
| **OVERALL PLANT-BASED DIET INDEX** | | | | | | | | | | | | |
| **Adjusting for aMED** | | | | | | | | | | | | |
| HRs for PDI | 1.00 | 0.99  (0.93, 1.05) | 0.92  (0.86, 0.98) | 0.93^b^  (0.87, 0.99) | 0.93  (0.87, 1.00) | 0.87  (0.81, 0.93) | 0.92  (0.85, 0.99) | 0.83  (0.77, 0.89) | 0.89  (0.82, 0.96) | 0.83  (0.77, 0.91) | 0.90  (0.87, 0.93) | <0.001 |
| HRs for aMED | 1.00 | 1.02  (0.95, 1.08) | 0.98  (0.91, 1.04) | 0.95  (0.89, 1.02) | 1.00  (0.93, 1.07) | 0.96  (0.90, 1.04) | 1.00  (0.93, 1.07) | 0.95  (0.88, 1.03) | 0.93  (0.86, 1.00) | 0.92  (0.85, 1.00) | 0.87  (0.77, 0.99) | 0.03 |
| **Adjusting for aHEI** | | | | | | | | | | | | |
| HRs for PDI | 1.00 | 0.99  (0.93, 1.06) | 0.93  (0.87, 0.99) | 0.94^b^  (0.88, 1.00) | 0.94  (0.88, 1.01) | 0.88  (0.82, 0.94) | 0.94  (0.87, 1.00) | 0.85  (0.79, 0.91) | 0.91  (0.85, 0.98) | 0.86  (0.80, 0.93) | 0.92  (0.89, 0.95) | <0.001 |
| HRs for aHEI | 1.00 | 0.91  (0.85, 0.96) | 0.95  (0.89, 1.01) | 0.89  (0.84, 0.95) | 0.89  (0.83, 0.95) | 0.85  (0.80, 0.91) | 0.85  (0.79, 0.91) | 0.74  (0.69, 0.80) | 0.80  (0.74, 0.86) | 0.72^b^  (0.67, 0.78) | 0.91^b^  (0.90, 0.93) | <0.001 |
| **Adjusting for DASH** | | | | | | | | | | | | |
| HRs for PDI | 1.00 | 1.01  (0.94, 1.07) | 0.94  (0.88, 1.01) | 0.96^b^  (0.90, 1.03) | 0.97  (0.90, 1.04) | 0.91  (0.85, 0.98) | 0.97  (0.90, 1.04) | 0.88  (0.82, 0.95) | 0.96  (0.89, 1.03) | 0.91  (0.84, 0.98) | 0.95  (0.91, 0.98) | 0.003 |
| HRs for DASH | 1.00 | 0.96  (0.90, 1.02) | 0.91  (0.86, 0.97) | 0.93  (0.87, 0.99) | 0.88  (0.82, 0.94) | 0.83^b^  (0.78, 0.89) | 0.80  (0.75, 0.86) | 0.82  (0.77, 0.88) | 0.73  (0.67, 0.78) | 0.74  (0.68, 0.80) | 0.79  (0.76, 0.83) | <0.001 |
| **HEALTHFUL PLANT-BASED DIET INDEX** | | | | | | | | | | | | |
| **Adjusting for aMED** | | | | | | | | | | | | |
| HRs for hPDI | 1.00 | 0.98  (0.92, 1.04) | 0.89  (0.84, 0.95) | 0.86^b^  (0.80, 0.92) | 0.81  (0.75, 0.87) | 0.81  (0.76, 0.87) | 0.81  (0.75, 0.87) | 0.73  (0.68, 0.79) | 0.71  (0.66, 0.77) | 0.63  (0.58, 0.69) | 0.81^b^  (0.78, 0.83) | <0.001^b^ |
| HRs for aMED | 1.00 | 1.04  (0.97, 1.10) | 1.01  (0.95, 1.08) | 1.00  (0.93, 1.07) | 1.06  (0.99, 1.14) | 1.04  (0.97, 1.12) | 1.09  (1.02, 1.18) | 1.07  (0.98, 1.15) | 1.05  (0.97, 1.14) | 1.08  (0.99, 1.18) | 1.19  (1.04, 1.36) | 0.03 |
| **Adjusting for aHEI** | | | | | | | | | | | | |
| HRs for hPDI | 1.00 | 1.01  (0.94, 1.07) | 0.93  (0.87, 0.99) | 0.90^b^  (0.84, 0.97) | 0.86  (0.80, 0.92) | 0.87  (0.81, 0.94) | 0.88  (0.81, 0.94) | 0.81  (0.75, 0.88) | 0.80  (0.73, 0.87) | 0.73  (0.66, 0.80) | 0.86  (0.83, 0.89) | <0.001 |
| HRs for aHEI | 1.00 | 0.92  (0.86, 0.97) | 0.97  (0.91, 1.04) | 0.93  (0.87, 0.99) | 0.94  (0.88, 1.00) | 0.91  (0.85, 0.98) | 0.92  (0.86, 0.99) | 0.82  (0.76, 0.89) | 0.90  (0.83, 0.98) | 0.85  (0.77, 0.93) | 0.96  (0.94, 0.98) | <0.001 |
| **Adjusting for DASH** | | | | | | | | | | | | |
| HRs for hPDI | 1.00 | 1.01  (0.95, 1.08) | 0.94  (0.88, 1.01) | 0.92^b^  (0.86, 0.99) | 0.88  (0.82, 0.95) | 0.90  (0.83, 0.96) | 0.90  (0.84, 0.98) | 0.84  (0.77, 0.91) | 0.83  (0.76, 0.90) | 0.76  (0.69, 0.83) | 0.88^b^  (0.85, 0.92) | <0.001 |
| HRs for DASH | 1.00 | 0.97  (0.91, 1.03) | 0.93  (0.87, 0.99) | 0.96  (0.90, 1.02) | 0.92  (0.86, 0.99) | 0.88^b^  (0.82, 0.95) | 0.86  (0.80, 0.93) | 0.89  (0.83, 0.96) | 0.80  (0.74, 0.87) | 0.85  (0.78, 0.93) | 0.87  (0.82, 0.91) | <0.001 |

*Results were pooled across the three cohorts using a fixed-effects model*

*Adjusted for age, smoking status, physical activity, alcohol intake, multivitamin use, family history of diabetes, margarine intake, energy intake, baseline hypertension, baseline hypercholesterolemia, and BMI. Also adjusted for menopause status and postmenopausal hormone use in NHS & NHS2, and for oral contraceptive use in NHS2.*

*^a^ p-Value when we assigned the median value to each decile and entered this as a continuous variable in the model*

*^b^ p-Value for Q-statistic for heterogeneity <0.05, indicating statistically significant heterogeneity in HRs among the three studies*

*Abbreviations: PDI, Overall Plant-based Diet Index; hPDI, Healthful Plant-based Diet Index; aMED, Alternate Mediterranean dietary pattern; aHEI, Alternate Healthy Eating Index; DASH, Dietary Approaches to Stop Hypertension*
