# Supplementary material for: Plant-Based Dietary Patterns and Incidence of Type 2 Diabetes in US Men and Women: Results from Three Prospective Cohort Studies
Source: PLoS Med. 2016 Jun 14;13(6):e1002039. doi: 10.1371/journal.pmed.1002039 (PMC4907448; doi:10.1371/journal.pmed.1002039)
Supplement: S1 Text — (DOCX) [file pmed.1002039.s011.docx]

**S1 Text:** STROBE Statement—checklist of items that should be included in reports of observational studies

|  | Item No. | Recommendation | Para  No. | Relevant text from manuscript |
| --- | --- | --- | --- | --- |
| **Title and abstract** | 1 | (*a*) Indicate the study’s design with a commonly used term in the title or the abstract | Title  Abstract, para 1 | : Results from Three Prospective Cohort Studies  …three prospective cohort studies in the US |
|  |  | (*b*) Provide in the abstract an informative and balanced summary of what was done and what was found | Abstract, para 1  Abstract, para 3 | We examined the association of an overall plant-based diet, and hypothesized healthful and unhealthful versions of a plant-based diet, with T2D incidence in three prospective cohort studies in the US…  In pooled multivariable-adjusted analysis, both the overall and healthful plant-based diet indices were inversely associated with T2D [(PDI: HR for extreme deciles, 0.51; 95% CI, 0.47-0.55; p trend<0.001) (hPDI: HR for extreme deciles, 0.55; 95% CI, 0.51-0.59; p trend<0.001)]. The association with PDI was considerably attenuated when we additionally adjusted for body mass index (BMI) categories (HR, 0.80; 95% CI, 0.74-0.87; p trend<0.001), while that with hPDI remained largely unchanged (HR, 0.66; 95% CI, 0.61-0.72; p trend<0.001). The unhealthful plant-based diet index was positively associated with T2D even after BMI adjustment (HR for extreme deciles, 1.16; 95% CI, 1.08-1.25; p trend<0.001). |
| Introduction | | | |  |
| Background/rationale | 2 | Explain the scientific background and rationale for the investigation being reported | Intro, para 1  Intro, para 2 | Type 2 diabetes (T2D) is associated with increased morbidity, mortality, and healthcare costs in the US.[1] Several plant foods, such as whole grains, fruits, and vegetables are associated with a lower risk of T2D,[2-4] while certain animal foods, such as red and processed meats, are positively associated with T2D risk.[5] Additionally, the recently released 2015 Dietary Guidelines Advisory Committee report recommends shifting away from intake of certain animal foods and moving towards a plant-rich diet.[6] Thus, we evaluated the hypothesis that a plant-based diet is protective against T2D.  Prior studies on plant-based diets and T2D[7-9] have defined them as ‘vegetarian’ diets, categorizing study populations dichotomously into participants who do or do not consume some or all animal foods. An important question from clinical and public health standpoints, however, is whether gradually moving towards a plant-rich diet by progressively decreasing animal food intake lowers T2D risk. If so, public health recommendations could suggest incremental dietary changes. Existing studies of vegetarian diets and T2D are also limited by a lack of differentiation among plant foods with divergent effects on T2D, because less nutrient-dense plant foods such as refined grains, potatoes, and sugar-sweetened beverages are associated with higher T2D risk.[10-12] |
| Objectives | 3 | State specific objectives, including any prespecified hypotheses | Intro, para 3 | We thus conceptualized a graded dietary pattern that positively weighs plant foods and negatively weighs animal foods,[13] and examined the association of this overall plant-based diet, and a priori, healthful and unhealthful versions of a plant-based diet, with T2D incidence in three large prospective cohort studies in the US. We hypothesized that these plant-based diets would be inversely associated with T2D risk. |
| Methods | | | |  |
| Study design | 4 | Present key elements of study design early in the paper | Intro, para 3 | …three large prospective cohort studies in the US |
| Setting | 5 | Describe the setting, locations, and relevant dates, including periods of recruitment, exposure, follow-up, and data collection | Methods, para 2 | The Nurses’ Health Study (NHS) started in 1976 with 121,701 female nurses (aged 30-55 years),[14] the NHS2 started in 1989 with 116,430 female nurses (aged 25-42 years),[15] and the Health Professionals Follow-up Study (HPFS) started in 1986 with 51,529 male health professionals (aged 40-75 years),[16] from across the US… In the current analysis, the 1984, 1986, and 1991 cycles were the baseline for NHS, NHS2, and HPFS respectively, when data on most covariates of interest were first comprehensively measured. |
| Participants | 6 | (*a*) *Cohort study*—Give the eligibility criteria, and the sources and methods of selection of participants. Describe methods of follow-up  *Case-control study*—Give the eligibility criteria, and the sources and methods of case ascertainment and control selection. Give the rationale for the choice of cases and controls  *Cross-sectional study*—Give the eligibility criteria, and the sources and methods of selection of participants | Methods, para 2 | Follow-up questionnaires collect information on lifestyle and medical history biennially, with a response rate of ~90% per cycle.  Participants with diabetes, cancer (except nonmelanoma skin cancer), cardiovascular disease (CVD), reported energy intake levels outside predefined limits (<600 or >3500 kcal/day for women and <800 or >4200 kcal/day for men), or incomplete dietary data at baseline were excluded. The final analysis included 69,949 women in NHS, 90,239 women in NHS2, and 40,539 men in HPFS at baseline. |
|  |  | (*b*) *Cohort study*—For matched studies, give matching criteria and number of exposed and unexposed  *Case-control study*—For matched studies, give matching criteria and the number of controls per case |  |  |
| Variables | 7 | Clearly define all outcomes, exposures, predictors, potential confounders, and effect modifiers. Give diagnostic criteria, if applicable | Methods, para 4-6  S1 Table  Methods, para 7 | Plant-based diet indices: We created an overall plant-based diet index (PDI), a healthful plant-based diet index (hPDI) an unhealthful plant-based diet index (uPDI)… The indices were analyzed as deciles, with energy intake adjusted at the analysis stage.  Definition of type 2 diabetes: Only confirmed cases that met ≥1 of the following criteria were included (as per the National Diabetes Data Group)… HbA1c≥6.5% was further added to the diagnosis criteria starting 2010.[25] |
| Data sources/ measurement | 8* | For each variable of interest, give sources of data and details of methods of assessment (measurement). Describe comparability of assessment methods if there is more than one group | Methods, para 3  Methods, para 7  Methods, para 8 | Dietary assessment: Dietary data were collected every 2-4 years using a semi-quantitative food frequency questionnaire… The reliability and validity of the questionnaires have been described previously.[17-20]  Ascertainment of type 2 diabetes: Participants who self-reported physician-diagnosed diabetes were sent a supplementary questionnaire with established validity to confirm diagnosis.[21, 22]  Assessment of covariates: We collected height at baseline and updated information on weight, physical activity, smoking, multivitamin use, ethnicity, family history of T2D, hypertension, and hypercholesterolemia through biennial questionnaires. In NHS and NHS2, we also assessed information on menopausal status, post-menopausal hormone use, and oral contraceptive use. |
| Bias | 9 | Describe any efforts to address potential sources of bias | Methods, para 9  Results, para 4 | We adjusted for smoking status, alcohol intake, physical activity, family history of diabetes, multivitamin use, margarine intake, energy intake, baseline hypertension and hypercholesterolemia, body mass index (BMI) categories, postmenopausal status & hormone use (women), and oral contraceptive use (NHS2).  Sensitivity analyses: Our findings remained robust in several sensitivity analyses. |
| Study size | 10 | Explain how the study size was arrived at | Methods, para 2 | Participants with diabetes, cancer (except nonmelanoma skin cancer), cardiovascular disease (CVD), reported energy intake levels outside predefined limits (<600 or >3500 kcal/day for women and <800 or >4200 kcal/day for men), or incomplete dietary data at baseline were excluded. The final analysis included 69,949 women in NHS, 90,239 women in NHS2, and 40,539 men in HPFS at baseline. |

Continued on next page

| Quantitative variables | 11 | Explain how quantitative variables were handled in the analyses. If applicable, describe which groupings were chosen and why | Methods, para 9 | For the primary analysis, we categorized the indices into deciles, so as to not make assumptions about linearity, and limit the influence of outlying observations… Continuous covariates were included in the model as categories for same the reasons cited above for categorizing the indices. |
| --- | --- | --- | --- | --- |
| Statistical methods | 12 | (*a*) Describe all statistical methods, including those used to control for confounding | Methods, para 9 | We calculated person-time for each participant from questionnaire return date until T2D diagnosis, death, censoring, or end of follow-up (30th June 2012 in NHS, 30th June 2011 in NHS2, and 1st January 2010 in HPFS). For the primary analysis, we categorized the indices into deciles, so as to not make assumptions about linearity, and limit the influence of outlying observations. We used Cox proportional-hazards regression to evaluate the associations between deciles of each index and T2D incidence. Age (years) was used as the time scale with stratification by calendar time (2-year intervals). We adjusted for smoking status, alcohol intake, physical activity, family history of diabetes, multivitamin use, margarine intake, energy intake, baseline hypertension and hypercholesterolemia, body mass index (BMI) categories, postmenopausal status & hormone use (women), and oral contraceptive use (NHS2). |
|  |  | (*b*) Describe any methods used to examine subgroups and interactions | Methods, para 10 | We tested for effect modification by age, physical activity, family history of diabetes, and BMI, by including cross-product terms. The analysis was carried out separately for each cohort, and combined using a fixed-effects model; the Cochrane Q statistic [26], the I2 statistic [27], and the between-study coefficient of variation [28,29] were used to assess heterogeneity among the cohorts. |
|  |  | (*c*) Explain how missing data were addressed | Methods, para 2 | [Participants with] …incomplete dietary data at baseline were excluded. |
|  |  | (*d*) *Cohort study*—If applicable, explain how loss to follow-up was addressed  *Case-control study*—If applicable, explain how matching of cases and controls was addressed  *Cross-sectional study*—If applicable, describe analytical methods taking account of sampling strategy | Methods, para 9 | We calculated person-time for each participant from questionnaire return date until T2D diagnosis, death, censoring, or end of follow-up (30th June 2012 in NHS, 30th June 2011 in NHS2, and 1st January 2010 in HPFS). |
|  |  | (*e*) Describe any sensitivity analyses | Results, para 4  Results, para 5  Results, para 6  Results, para 7 | Our findings remained robust in several sensitivity analyses … Stratified analysis showed no significant effect modification by ethnicity for the diet indices.  To examine the individual contributions of healthy plant, less healthy plant, and animal foods to T2D risk, we included variables for all three food groups simultaneously in the fully-adjusted model…  To examine the effect of consuming a healthful plant-based diet that is also high in intake of some animal foods known to be associated with reduced risk of several health outcomes (e.g. fish and yogurt intake [30-33]) …  Previous analysis in these cohorts have found other dietary patterns such as the Mediterranean diet (aMED), the alternate Healthy Eating Index (aHEI), and Dietary Approaches to Stop Hypertension (DASH), to be inversely associated with T2D [34-36] … |
| Results | | | | |
| Participants | 13* | (a) Report numbers of individuals at each stage of study—eg numbers potentially eligible, examined for eligibility, confirmed eligible, included in the study, completing follow-up, and analysed | Methods, para 2 | The Nurses’ Health Study (NHS) started in 1976 with 121,701 female nurses (aged 30-55 years),[14] the NHS2 started in 1989 with 116,430 female nurses (aged 25-42 years),[15] and the Health Professionals Follow-up Study (HPFS) started in 1986 with 51,529 male health professionals (aged 40-75 years),[16] from across the US… The final analysis included 69,949 women in NHS, 90,239 women in NHS2, and 40,539 men in HPFS at baseline. |
|  |  | (b) Give reasons for non-participation at each stage |  |  |
|  |  | (c) Consider use of a flow diagram |  |  |
| Descriptive data | 14* | (a) Give characteristics of study participants (eg demographic, clinical, social) and information on exposures and potential confounders | Results, para 1  Table 1 and S2 Table  Results, para 2 | Baseline characteristics: Participants with higher scores on the PDI or hPDI were older, more active, leaner, and less likely to smoke than participants with lower scores (Table 1 and S2 Table). They also consumed a lower percentage of calories from saturated and monounsaturated fats, a higher percentage of calories from polyunsaturated fats and carbohydrates, and higher levels of fiber and folate.  During 4,102,369 person-years of follow-up, we documented 16,162 T2D cases. |
|  |  | (b) Indicate number of participants with missing data for each variable of interest |  |  |
|  |  | (c) *Cohort study*—Summarise follow-up time (eg, average and total amount) |  |  |
| Outcome data | 15* | *Cohort study*—Report numbers of outcome events or summary measures over time | Results, para 2 | During 4,102,369 person-years of follow-up, we documented 16,162 T2D cases. |
|  |  | *Case-control study—*Report numbers in each exposure category, or summary measures of exposure |  |  |
|  |  | *Cross-sectional study—*Report numbers of outcome events or summary measures |  |  |
| Main results | 16 | (*a*) Give unadjusted estimates and, if applicable, confounder-adjusted estimates and their precision (eg, 95% confidence interval). Make clear which confounders were adjusted for and why they were included | Results, para 2  Results, para 3  Tables 2 & 3, and Fig 1 | PDI was inversely associated with T2D incidence in all three cohorts after adjusting for potential confounders (Table 2) …  After multivariable adjustment, a strong inverse association was observed between hPDI and T2D (Table 3), which was only modestly attenuated after BMI adjustment… In contrast, the unhealthful plant-based diet index was positively associated with T2D (pooled HR for extreme deciles, 1.16; 95% CI, 1.08-1.25; p trend<0.001) (Fig 1). |
|  |  | (*b*) Report category boundaries when continuous variables were categorized |  |  |
|  |  | (*c*) If relevant, consider translating estimates of relative risk into absolute risk for a meaningful time period |  |  |

Continued on next page

| Other analyses | 17 | Report other analyses done—eg analyses of subgroups and interactions, and sensitivity analyses | Results, para 4-7  Fig 2, S1-S3 Figures, and S3-S7 Tables | Sensitivity analyses: Our findings remained robust in several sensitivity analyses… Pooled HRs for both PDI and hPDI remained largely unchanged when the Mediterranean diet was controlled for, and were only slightly attenuated with the alternate Healthy Eating Index or Dietary Approaches to Stop Hypertension in the same model. |
| --- | --- | --- | --- | --- |
| Discussion | | | | |
| Key results | 18 | Summarise key results with reference to study objectives | Discussion, para 1 | We found significant, linear, inverse associations of plant-based diets, especially a healthier version (hPDI), with T2D incidence in three prospective cohorts in the US. In contrast, a less healthy version of a plant-based diet (uPDI) was associated with increased T2D risk. These associations were independent of BMI and other diabetes risk factors. |
| Limitations | 19 | Discuss limitations of the study, taking into account sources of potential bias or imprecision. Discuss both direction and magnitude of any potential bias | Discussion, para 6 | Our study has several limitations… |
| Interpretation | 20 | Give a cautious overall interpretation of results considering objectives, limitations, multiplicity of analyses, results from similar studies, and other relevant evidence | Conclusions | Conclusions: We found an inverse association between an overall plant-based diet and T2D incidence in three prospective cohorts. This inverse association became substantially stronger for a healthier version of the diet, but was positive for an unhealthful version. Our study supports current recommendations to shift to diets rich in healthy plant foods, with lower intake of less healthy plant and animal foods. |
| Generalisability | 21 | Discuss the generalisability (external validity) of the study results | Discussion, para 6  S2 Fig | Given that we found similar associations between the plant-based diet indices and T2D among different ethnic groups, it is likely that these findings are generalizable to diverse racial/ethnic groups. Nevertheless, these studies were carried out among health professionals in the US, and hence it would be important to replicate these findings in other populations representing diverse countries and occupational groups. |
| Other information | |  | | |
| Funding | 22 | Give the source of funding and the role of the funders for the present study and, if applicable, for the original study on which the present article is based | Funding section | Funding/Support: This work was supported by research grants DK58845, UM1 CA186107, UM1 CA176726, and UM1 CA167552 from the National Institutes of Health.  Role of the Funder/Sponsor: The funders had no role in study design, data collection and analysis, decision to publish, or preparation of the manuscript. |

*Give information separately for cases and controls in case-control studies and, if applicable, for exposed and unexposed groups in cohort and cross-sectional studies.

**Note:** An Explanation and Elaboration article discusses each checklist item and gives methodological background and published examples of transparent reporting. The STROBE checklist is best used in conjunction with this article (freely available on the Web sites of PLoS Medicine at http://www.plosmedicine.org/, Annals of Internal Medicine at http://www.annals.org/, and Epidemiology at http://www.epidem.com/). Information on the STROBE Initiative is available at www.strobe-statement.org.
