# Supplementary material for: Plant-Based Dietary Patterns and Incidence of Type 2 Diabetes in US Men and Women: Results from Three Prospective Cohort Studies
Source: PLoS Med. 2016 Jun 14;13(6):e1002039. doi: 10.1371/journal.pmed.1002039 (PMC4907448; doi:10.1371/journal.pmed.1002039)
Supplement: S2 Text — (DOCX) [file pmed.1002039.s012.docx]

**S2 Text: Prospective Analysis Plan**

**Analysis proposal: Vegetarian-Style Diet Index & Type 2 Diabetes**

**Lead author:** Ambika Satija

**Main senior author:** Frank B Hu

**Other co-authors (alphabetical):** Shilpa Bhupathiraju, Walter Willett

**Purpose:**

To develop vegetarian-style diet indices (VDIs), high adherence to which implies high intake of some/all plant source-foods and low intake of animal source-foods, and prospectively examine their association with type 2 diabetes. Two such VDIs will be created: 1) total VDI, high adherence to which implies high intake of all plant source-foods and low intake of animal source-foods, and 2) alternate VDI, high adherence to which implies high intake of healthy plant source-foods and low intake of unhealthy plant-source foods and low intake of animal source-foods.

**Background:**

Type 2 diabetes (T2D) is associated with high morbidity, mortality, and healthcare costs in the US and throughout the world. Dietary factors have been linked with T2D – many plant-source foods have been associated with decreased risk of T2D, and some animal-source foods with increased risk of T2D. This raises the question of whether a diet high in plant-source foods and low in animal-source foods is protective of diabetes. As it is possible to consume a plant-based diet that is predominantly high in unhealthy plant-source foods (e.g. sugar sweetened beverages, or SSB, refined grains), it is important to ascertain whether in addition to an overall plant-based diet, a healthy plant-based diet is associated with reduced risk of diabetes.

Numerous studies have found an inverse association between plant-based diets and several health outcomes, but these studies have assessed plant-based diets as “vegan/vegetarian” diets, defined such diets in varying ways, and not made a distinction between healthy and unhealthy foods. In addition, very few studies have prospectively examined T2D as an outcome.

Thus, the proposed study aims to fill this gap in knowledge by creating a vegetarian-style diet index and an alternate vegetarian-style diet index in a standardized manner using FFQ data from three large US cohorts, and assessing their associations with 20-year T2D incidence.

**Study Population:**

NHSI 1984-2008

NHSII 1991-2009

HPFS 1986-2008

**Outcome:**

Type 2 Diabetes (only confirmed cases)

**Exposures:**

1. Total Vegetarian-style Diet Index (to construct)
2. Alternate Vegetarian-style Diet Index (to construct)

All dietary measures will be cumulatively averaged, but updating will stop upon the development of intermediate outcomes.

**Covariates:**

Smoking, physical activity, alcohol consumption, postmenopausal hormone use, multivitamin use, family history of diabetes, diet beverage intake, body mass index, total energy intake, hypertension history and hypercholesterolemia history

**Analyses:**

Cox proportional hazards models, conditioned on age and follow-up cycle.
